# *Trypanosoma brucei* infection protects mice against malaria

**DOI:** 10.1371/journal.ppat.1008145

**Published:** 2019-11-08

**Authors:** Margarida Sanches-Vaz, Adriana Temporão, Rafael Luis, Helena Nunes-Cabaço, António M. Mendes, Sarah Goellner, Tânia Carvalho, Luisa M. Figueiredo, Miguel Prudêncio

**Affiliations:** Instituto de Medicina Molecular João Lobo Antunes, Faculdade de Medicina, Universidade de Lisboa, Lisboa, Portugal; Queensland Institute of Medical Research, AUSTRALIA

## Abstract

Sleeping sickness and malaria are parasitic diseases with overlapping geographical distributions in sub-Saharan Africa. We hypothesized that the immune response elicited by an infection with *Trypanosoma brucei*, the etiological agent of sleeping sickness, would inhibit a subsequent infection by *Plasmodium*, the malaria parasite, decreasing the severity of its associated pathology. To investigate this, we established a new co-infection model in which mice were initially infected with *T*. *brucei*, followed by administration of *P*. *berghei* sporozoites. We observed that a primary infection by *T*. *brucei* significantly attenuates a subsequent infection by the malaria parasite, protecting mice from experimental cerebral malaria and prolonging host survival. We further observed that an ongoing *T*. *brucei* infection leads to an accumulation of lymphocyte-derived IFN-γ in the liver, limiting the establishment of a subsequent hepatic infection by *P*. *berghei* sporozoites. Thus, we identified a novel host-mediated interaction between two parasitic infections, which may be epidemiologically relevant in regions of *Trypanosoma*/*Plasmodium* co-endemicity.

## Author summary

Despite the geographical overlap between the parasites that cause sleeping sickness and malaria, the reciprocal impact of a co-infection by *T*. *brucei* and *Plasmodium* had hitherto not been assessed. We hypothesized that the strong immune response elicited by a *T*. *brucei* infection could potentially limit the ability of *Plasmodium* parasites to infect the same host. In this study, we showed that a primary infection by *T*. *brucei* significantly attenuates a subsequent infection by the malaria parasite. Importantly, a significant proportion of the co-infected mice do not develop *Plasmodium* parasitemia, and those few that do, do not display symptoms of severe malaria and survive longer than their singly infected counterparts. We further showed that the prevention or delay in appearance of malaria parasites in the blood results from a dramatic impairment of the preceding liver infection by *Plasmodium*, which is mediated by the strong immune response mounted against the primary *T*. *brucei* infection. Our study provides new insights for a novel inter-pathogen interaction that may bear great epidemiological significance in regions of *Trypanosoma*/*Plasmodium* co-endemicity.

## Introduction

The term co-infection refers to a condition in which a host is concomitantly infected by two or more infectious agents [1]. It is estimated that co-infections occur in over one sixth of the world’s human population [2]. The outcome of the interaction between two or more co-infecting agents may be complex. It may result in either the enhancement or the suppression of the growth of one or both pathogen(s), and may lead to the aggravation or improvement of the pathology of either infection [1].

Co-infections between the malaria parasite and other pathogens are highly prevalent in Sub-Saharan Africa [3–6]. Malaria is a disease caused by intracellular *Plasmodium* parasites, which infect their mammalian hosts through the bite of infected female *Anopheles* mosquitoes during a blood meal. Inoculated sporozoites reach the blood circulation and home to the liver, where they cross the endothelium of sinusoids and traverse several hepatocytes before productively infecting a final one. Inside the hepatocyte, sporozoites differentiate into intrahepatic or exoerythrocytic forms (EEF) that replicate extensively, originating thousands of merozoites. At the end of the obligatory but asymptomatic liver stage of infection, merozoites are released into the bloodstream, infecting red blood cells (RBCs) and leading to pathology [7].

Sub-Saharan African populations are also affected by human African trypanosomiasis (HAT), or sleeping sickness, a neglected tropical disease caused by extracellular *Trypanosoma brucei* parasites. Upon a blood meal by the *Glossina* fly, trypanosomes are released into the skin of the mammalian host, rapidly reaching and replicating in the blood, and eventually colonizing other organs, such as the brain, skin and adipose tissue [8, 9]. During an infection by *T*. *brucei*, the host’s innate immune system encounters several parasite antigens, such as the variant surface glycoprotein (VSG) and the trypanosome-lymphocyte triggering factor (TLTF) [10]. The former is usually associated with the activation of both T and B lymphocytes, as well as macrophages, leading to a pro-inflammatory Th1 response, characterized by the production of several cytokines, including IFN-γ, TNF-α and IL-6, as well as nitric oxide. The latter further contributes to the production of IFN-γ through the early activation of natural killer cells [11].

In humans, concomitant infections by *Trypanosoma* and *Plasmodium* parasites have been reported in regions where malaria and HAT are co-endemic [12–19]. However, the impact of these co-infections on pathology has only been assessed in two clinical studies [16, 19]. A cross-sectional study in Southern Sudan showed that trypanosome-infected patients presented a reduced number of white blood cells, while that number was increased in co-infected patients [19]. In contrast, no major differences were observed in the clinical and treatment outcomes of *Trypanosoma*/*Plasmodium* co-infected patients in Tanzania and Uganda, relative to those infected by *T*. *b*. *rhodesiense* only [16]. To the best of our knowledge, only two studies have investigated *Trypanosoma*/*Plasmodium* co-infections experimentally, using mouse models of infection [20, 21]. The first study showed that when erythrocytic stages of *P*. *chabaudi* were inoculated in mice with an ongoing *T*. *brucei* infection, the onset of *Plasmodium* parasitemia was delayed but rapidly reached a peak equivalent to that observed in the absence of trypanosomes [20]. A more recent study reported that mice simultaneously infected with *T*. *brucei* and *P*. *berghei* display higher parasitemia for both pathogens, leading to a significant decrease in host survival relative to mice infected with either one of these parasites alone [21].

Given that *T*. *brucei* elicits the production of IFN-γ, a cytokine that has been previously shown to inhibit the development of *Plasmodium* parasites [22–24], we postulated that a *T*. *brucei* infection might influence the host’s susceptibility to the malaria parasite. To test this hypothesis, a co-infection protocol employing rodent *T*. *brucei* and *P*. *berghei* parasites, two well-established models for sleeping sickness and malaria, respectively [8, 25], was designed to investigate *in vivo* the impact of an ongoing *T*. *brucei* infection on a subsequent infection by *P*. *berghei*. We observed that a primary infection by *T*. *brucei* significantly attenuates a subsequent infection by the malaria parasite, protecting mice from experimental cerebral malaria and improving host survival, relative to mice infected only with *Plasmodium*. Our results further show that an ongoing infection by *T*. *brucei* leads to a significant increase of IFN-γ levels in the host’s liver, which dramatically inhibits a subsequent hepatic infection by *P*. *berghei*.

## Results

### *T*. *brucei* infection attenuates a subsequent infection by the malaria parasite

**Fig 1:**
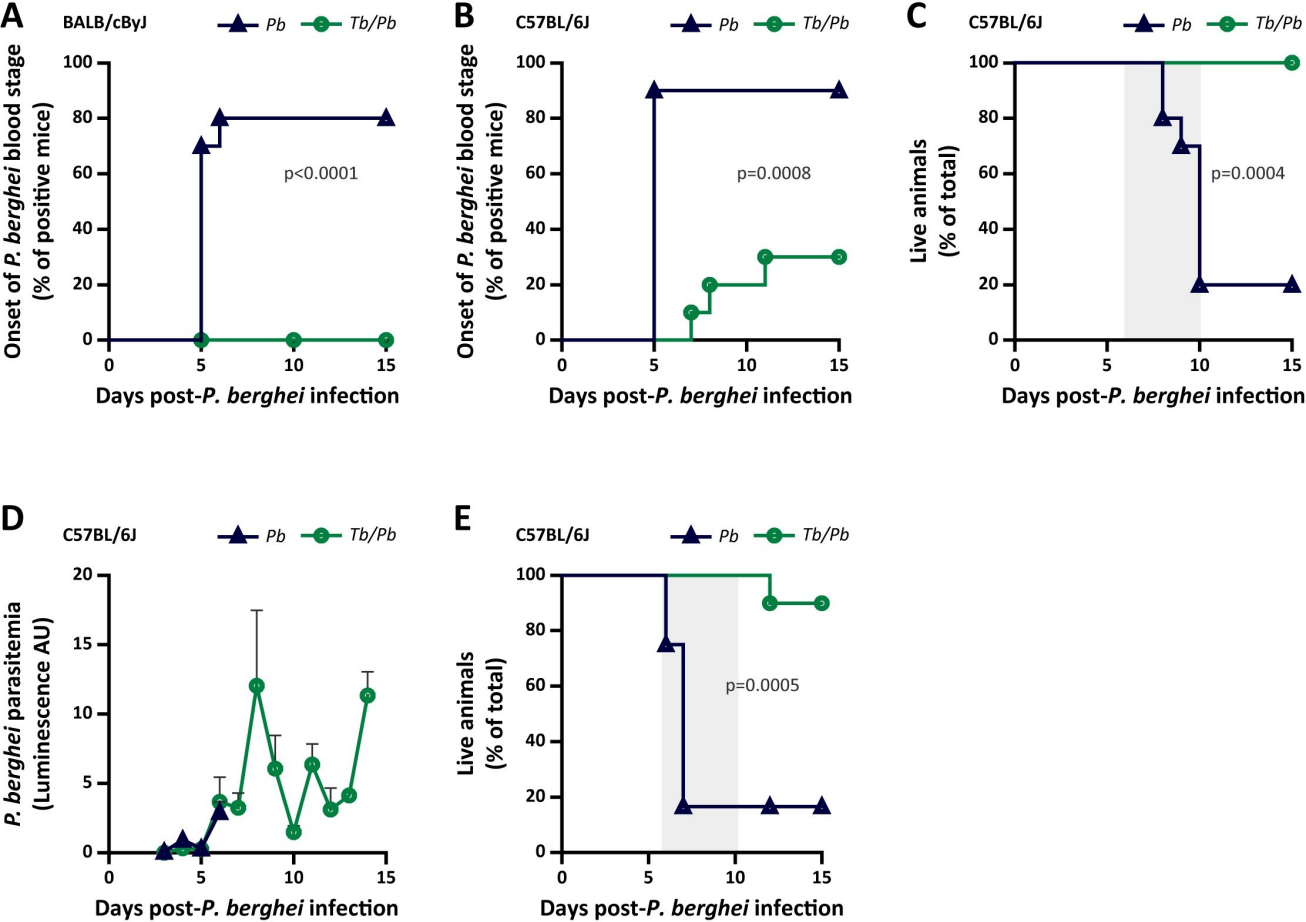
*T*. *brucei* infection protects mice from malaria. **(A)** Assessment of *P*. *berghei* prepatency period following inoculation of 500 *P*. *berghei* sporozoites into naïve BALB/cByJ mice (*Pb*—blue line) or BALB/cByJ mice infected 5 days earlier with *T*. *brucei* (*Tb/Pb*—green line). Percentage of mice displaying *P*. *berghei* parasitemia, as measured by flow cytometry. The pooled data of 10 mice from in two independent experiments is shown. **(B)** Assessment of *P*. *berghei* prepatency period following inoculation of 500 *P*. *berghei* sporozoites into naïve C57BL/6J mice (*Pb*—blue line) or C57BL/6J mice infected 5 days earlier with *T*. *brucei* (*Tb/Pb*—green line). Percentage of mice displaying *P*. *berghei* parasitemia, as measured by flow cytometry. The pooled data of 10 mice from two independent experiments is shown. **(C)** Mouse survival following inoculation of 500 *P*. *berghei* sporozoites into naïve C57BL/6J mice (*Pb*—blue line) or C57BL/6J mice infected 5 days earlier with *T*. *brucei* (*Tb/Pb*—green line). Percentage of live mice from a pool of 10 mice employed in two independent experiments. **(D)** Assessment of *P*. *berghei* parasitemia after inoculation of 1 x 10^6^ iRBCs into naïve C57BL/6J mice (*Pb*—blue line) or C57BL/6J mice infected 5 days earlier with *T*. *brucei* (*Tb/Pb*—green line). The mean bioluminescence and SEM of the pooled data of 10 mice from two independent experiments is shown. **(E)** Mouse survival following inoculation of 1 x 10^6^ iRBCs into naïve C57BL/6J mice (*Pb*—blue line) or C57BL/6J mice infected 5 days earlier with *T*. *brucei* (*Tb/Pb*—green line). Percentage of live mice from a pool of 10 mice from two independent experiments. For **A** and **B**, the Mantel-Cox (log rank) test was employed to compare the onset of *P*. *berghei* parasitemia, indicating statistically significant differences for the *Tb/Pb* group compared to the *Pb* control group. For **C** and **E**, the Mantel-Cox (log rank) test was employed to compare survival curves, indicating statistically significant differences for *Tb/Pb* compared to the *Pb* control, and the time window for ECM development is depicted by the grey-shaded area.

To assess whether the outcome of an infection with the malaria parasite is affected by an ongoing *T*. *brucei* infection, BALB/cByJ or C57BL/6J mice were initially infected with *T*. *brucei* and, five days later, exposed to the bites of five *P*. *berghei*-infected *Anopheles* mosquitoes, the lowest dose previously shown to warrant infection of 100% of the exposed mice [26]. Having observed a high intra- and inter-experimental variability in the number of mosquitoes that ingest a blood meal, as well as different biting preferences for *T*. *brucei*-infected and uninfected mice (**S1 Fig**), we subsequently infected animals by i.v. injection of 500 GFP-expressing *P*. *berghei* sporozoites, employed as a surrogate of the inoculum delivered by the bites of five infected mosquitoes [7]. Whereas 80% of the mice infected only with *P*. *berghei* developed an erythrocytic infection within 5 to 6 days after sporozoite inoculation, none of the co-infected BALB/cByJ mice presented *P*. *berghei* parasitemia for up to 15 days after sporozoite administration (**Figs 1A** and **S2A**). C57BL/6J mice are typically more susceptible to hepatic infection by *P*. *berghei* and constitute a good model for experimental cerebral malaria (ECM) [27, 28]. Strikingly, when a similar experimental setup was employed in the C57BL/6J mouse strain, 70% of the co-infected mice did not display a *P*. *berghei* erythrocytic infection for up to 15 days after sporozoite administration, whereas only 1 out of 10 mice infected with *P*. *berghei* alone did not develop parasitemia (**Figs 1B** and **S2B**). We further observed that the prepatent period in the few C57BL/6J mice that did develop an erythrocytic infection was at least two days longer in co-infected mice than in mice infected only with *P*. *berghei*. In a *Plasmodium* infection, the duration of the prepatent period has been described as a good predictor for the amount of infective merozoites that exit the liver, which directly depends on the total hepatic parasite load [29]. Therefore, our results suggest that an initial infection with *T*. *brucei* leads to a strong reduction in *P*. *berghei* hepatic burden in the co-infected mice, which either prevents the appearance of *Plasmodium* parasitemia or significantly delays its onset.

The *Trypanosoma/Plasmodium* co-infection had a major impact on malaria severity. In fact, whereas ~90% of the blood stage-positive control C57BL/6J mice succumbed within 8 to 10 days after sporozoite administration with signs of ECM, none of the co-infected mice that developed parasitemia displayed ECM symptoms or died within that time frame (**Fig 1C**). It has been reported that a delay in the onset of *P*. *berghei* parasitemia in C57BL/6J mice is associated with protection against cerebral pathology and increased host survival [30–33]. Thus, the absence of ECM in co-infected C57BL/6J mice could be due to the delay in the onset of *P*. *berghei* parasitemia and/or to a direct impact of the *T*. *brucei* infection on *Plasmodium* erythrocytic development. In order to assess these possible scenarios experimentally, the liver stage of the *Plasmodium* life cycle was bypassed by inoculating *P*. *berghei*-iRBCs into *T*. *brucei*-infected or naïve mice, and both groups of animals were monitored for the ensuing parasitemia and disease symptoms. Our results show that although the replication of *P*. *berghei* blood-stages is not compromised by an ongoing trypanosome infection (**Fig 1D**), co-infected mice did not develop ECM symptoms and displayed a significant increase in survival, compared with *P*. *berghei* single-infected mice (**Fig 1E**), indicating that *T*. *brucei* can directly attenuate *Plasmodium* erythrocytic infection, in a liver-independent manner. Of note, *T*. *brucei* parasitemia was unaffected by the injection of *P*. *berghei*-iRBCs on day 5 of trypanosome infection, indicating that *T*. *brucei* replication in the blood is not affected by *P*. *berghei* (**S3 Fig**).

Altogether, our data show that a primary infection by *T*. *brucei* renders mice more resistant to a subsequent infection by *P*. *berghei*, strongly reducing malaria pathology, decreasing disease severity, and improving host survival relative to mice infected only with *Plasmodium*.

### *T*. *brucei* infection reduces a subsequent liver infection by *P*. *berghei*

**Fig 2:**
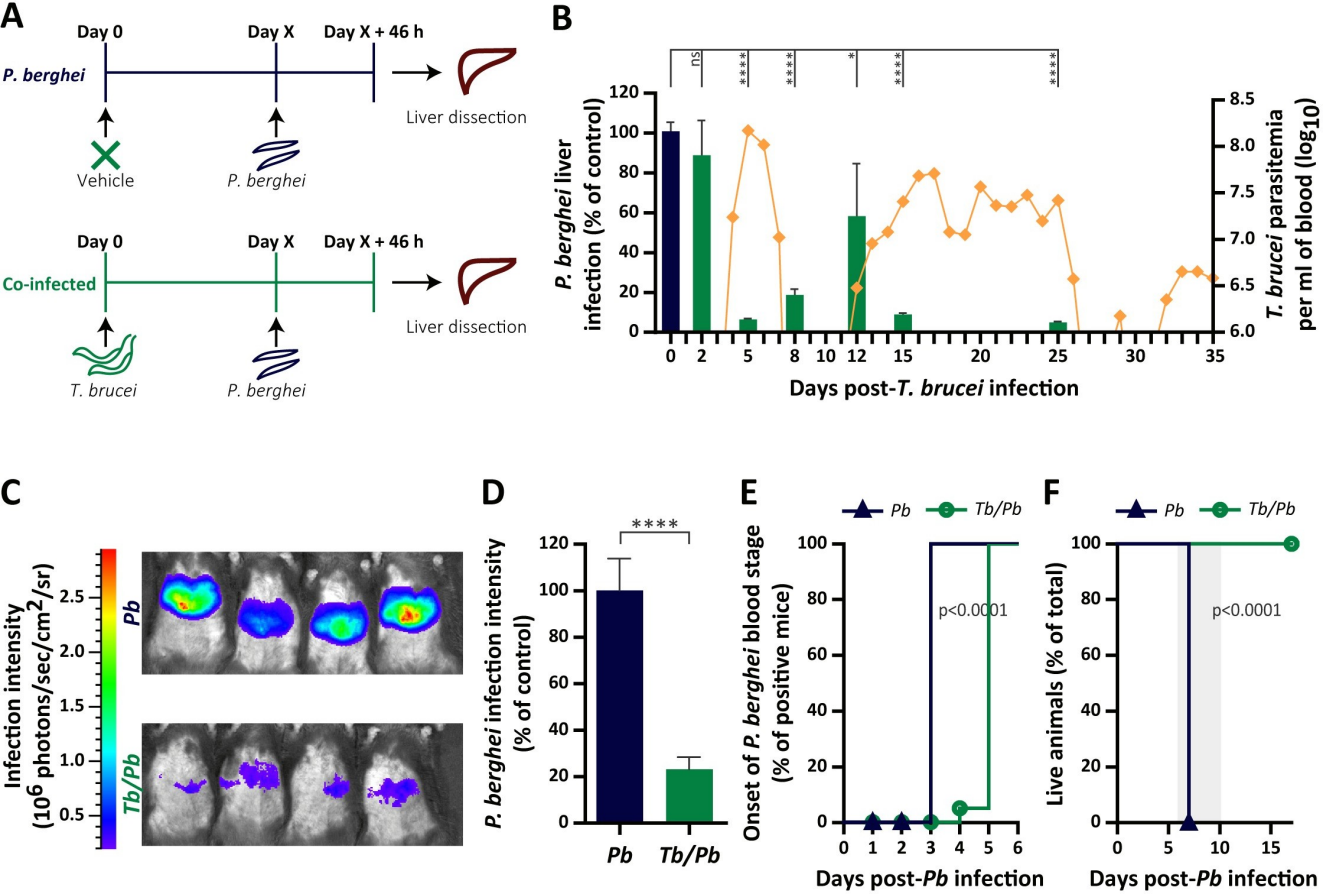
*T*. *brucei* attenuates hepatic infection by *P*. *berghei*. **(A)** Schematic illustration of the co-infection experimental design. The arrows indicate the times of *T*. *brucei* and/or *P*. *berghei* inoculation, and of liver dissection for quantification of *P*. *berghei* liver load. **(B)** *P*. *berghei* liver infection load (bars–primary YY axis) determined by qRT-PCR 46 h after sporozoite injection into naïve mice (blue bar) or mice previously infected by *T*. *brucei* (green bars), and *T*. *brucei* parasitemia (dots–secondary YY axis) determined daily by microscopy. All *P*. *berghei* infections were performed on the same day, with prior *T*. *brucei* infections staggered to give a consistent day for subsequent *P*. *berghei* infection. The time points indicated on the XX-axis correspond to the number of days that elapsed between *T*. *brucei* inoculation and sporozoite injection. Bars represent the mean values of two independent experiments and error bars indicate the SEM. The one-way ANOVA with post-test Dunnett was employed to assess the statistical significance of differences between the experimental groups. ns, not significant, * *P* < 0.05 and **** *P* < 0.0001. **(C)** Representative bioluminescence images of mouse livers 46 h after inoculation of 3 x 10^4^ *P*. *berghei* sporozoites into either naïve mice (*Pb*—top) or mice infected 5 days earlier with *T*. *brucei* (*Tb/Pb*—bottom). **(D)** Quantification of the *P*. *berghei* liver infection load measured by bioluminescence 46 h after sporozoite injection into naïve mice (blue bar) or mice previously infected by *T*. *brucei* (green bar). Bars represent the mean values of three independent experiments and error bars indicate the SEM. The Mann-Whitney test was employed to assess the statistical significance of differences between experimental groups (**** *P* < 0.0001). **(E)** Assessment of *P*. *berghei* prepatency period following inoculation of 3 x 10^4^ sporozoites into naïve mice (*Pb*—blue line) or mice infected 5 days beforehand with *T*. *brucei* (*Tb/Pb*—green line). Percentage of mice displaying *P*. *berghei* parasitemia, as measured by flow cytometry. The pooled data from 10 mice employed in two independent experiments is shown The Mantel-Cox (log rank) test was employed to compare the onset of *P*. *berghei* parasitemia curves, indicating statistically significant differences for *Tb/Pb* compared to the *Pb* control. **(F)** Mouse survival following inoculation of 3 x 10^4^ *P*. *berghei* sporozoites into naïve C57BL/6J mice (*Pb*—blue line) or C57BL/6J mice infected 5 days earlier with *T*. *brucei* (*Tb/Pb*—green line). Percentage of live mice from a pool of 10 mice employed in two independent experiments. The Mantel-Cox (log rank) test was employed to compare survival curves, indicating statistically significant differences for *Tb/Pb* compared to the *Pb* control, and the time window for ECM development is depicted by the grey-shaded area.

Having observed that a primary infection by *T*. *brucei* prevents or delays the appearance of *P*. *berghei* parasitemia in co-infected mice, we investigated the impact of an ongoing *T*. *brucei* infection on the liver stage of the *Plasmodium* life cycle. To this end, 3 x 10^4^ *P*. *berghei* sporozoites, a dose that enables an accurate quantification of hepatic infection, were inoculated into C57BL/6J mice infected with *T*. *brucei* parasites 2 to 25 days earlier (**Fig 2A**). Forty-six hours after sporozoite injection, the *P*. *berghei* liver load was determined by quantitative real-time reverse transcriptase-PCR (qRT-PCR) and compared with that of mice infected with *P*. *berghei* only (**Fig 2B**). Our results show that if *P*. *berghei* sporozoites were inoculated 2 days after *T*. *brucei* infection, when trypanosomes are not yet detectable in the blood, co-infected mice displayed a *P*. *berghei* liver load similar to that observed in single-infected mice. However, when sporozoites were injected 5, 8, 12, 15 or 25 days after *T*. *brucei* infection, co-infected mice showed a striking ~82–96% reduction in *P*. *berghei* liver load compared to control mice (**Fig 2B**). A similar reduction (~85%) was detected when mice were co-infected with *T*. *brucei* and *P*. *yoelli*, which indicates that *T*. *brucei* impairs the hepatic infection by several *Plasmodium* species (**S4 Fig**).

In view of these results, *P*. *berghei* sporozoites were inoculated 5 days after the initial *T*. *brucei* infection in all subsequent co-infection experiments. To further confirm the inhibition of *P*. *berghei* liver infection by a primary *T*. *brucei* infection, we administered luciferase-expressing *P*. *berghei* sporozoites into naïve mice and into mice infected 5 days earlier with *T*. *brucei*, and subsequently assessed the hepatic infection in both groups of mice 46 h later by bioluminescence. In agreement with our qRT-PCR results, co-infected mice displayed a ~80% reduction in *P*. *berghei* liver burden, as measured by luciferase activity, when compared to control mice (**Fig 2C** and **2D**). Moreover, when *P*. *berghei* infection was allowed to proceed to the blood, although all co-infected mice eventually developed *Plasmodium* parasitemia, they displayed a 2-day increase in prepatency period relative to single-infected mice (**Fig 2E**). The delay in the onset of *P*. *berghei* parasitemia observed in co-infected mice confirms a marked decrease in the number of infective merozoites that exit the liver of these mice, relative to their counterparts infected only with *Plasmodium*. Importantly, all the co-infected mice survived for longer than 17 days and did not present ECM symptoms, whereas infected control mice succumbed within 7 days after sporozoite administration with signs of ECM (**Fig 2F**).

To assess whether the observed protection against *Plasmodium* erythrocytic infection and ECM is time-dependent, *P*. *berghei* sporozoites were injected into mice 15 days after *T*. *brucei* inoculation, employing *P*. *berghei* single-infected mice as controls, and infection was allowed to proceed to the blood. We observed that co-infected mice displayed a 2-day delay in the appearance of *P*. *berghei* parasitemia and did not develop ECM symptoms (**S5 Fig**). These results are similar to those obtained for mice infected with *P*. *berghei* sporozoites 5 days after the initial *T*. *brucei* infection (**Fig 2E**), indicating that *T*. *brucei*-mediated protection against blood stage *Plasmodium* infection and ECM is not time-restricted.

Altogether, our data show that a primary infection by *T*. *brucei* not only attenuates the severity of the blood stage of *P*. *berghei* infection but also significantly reduces its hepatic infection, the first stage of *Plasmodium* life cycle in the mammalian host.

### *T*. *brucei* infection reduces the number of *P*. *berghei*-infected hepatocytes

**Fig 3:**
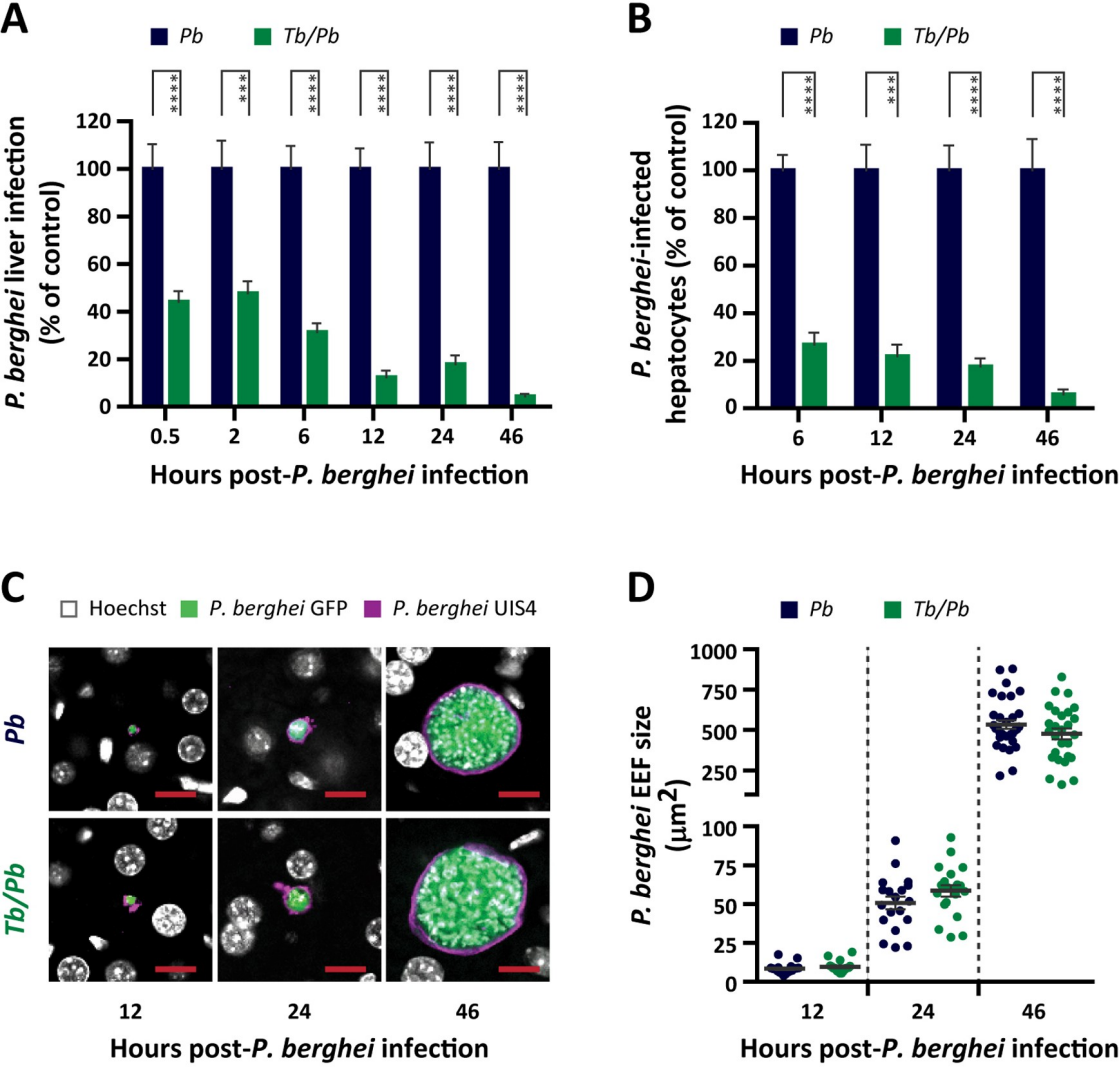
*T*. *brucei* impairs early liver stage of *P*. *berghei* infection. **(A)** *P*. *berghei* liver infection load quantified by qRT-PCR 30 min, 2 h, 6 h, 12 h, 24 h and 46 h after injection of 3 x 10^4^ *P*. *berghei* sporozoites into naïve mice (*Pb*–blue bars) or mice infected 5 days earlier with *T*. *brucei* (*Tb/Pb*–green bars). Bars represent the mean values of three to five independent experiments and error bars indicate the SEM. The two-way ANOVA with post-test Bonferroni was employed to assess the statistical significance of differences between experimental groups. *** *P* < 0.001 and **** *P* < 0.0001. **(B)** Number of *P*. *berghei*-infected hepatocytes per square millimeter of liver section quantified by immunofluorescence microscopy 6 h, 12 h, 24 h and 46 h after injection of 3 x 10^4^ *P*. *berghei* sporozoites into naïve mice (*Pb*–blue bars) or mice infected 5 days earlier with *T*. *brucei* (*Tb/Pb*–green bars). Bars represent the mean values of one representative experiment out of two independent experiments and error bars indicate the SEM. The two-way ANOVA with post-test Bonferroni was employed to assess the statistical significance of differences between experimental groups. *** *P* < 0.001 and **** *P* < 0.0001. **(C)** Representative confocal microscopy images of EEFs at 12 h, 24 h and 46 h after injection of 3 x 10^4^ *P*. *berghei* sporozoites into naïve mice or mice infected 5 days earlier with *T*. *brucei*. White: Hoechst—nuclear staining; green: *P*. *berghei* GFP labeling showing the parasite heat shock protein 70; purple: *P*. *berghei* UIS4 labeling showing the parasitophorous vacuole membrane. Scale bars, 10 μm. **(D)** EEF area at 12 h, 24 h and 46 h after injection of 3 x 10^4^ *P*. *berghei* sporozoites into naïve mice (*Pb*—blue dots) or mice infected 5 days earlier with *T*. *brucei* (*Tb/Pb*—green dots), assessed by immunofluorescence microscopy. Results are expressed as the mean values of one representative experiment out of two independent experiments and error bars indicate the SEM.

The remarkable impairment of *P*. *berghei* liver infection by *T*. *brucei*, and the fact that the liver stage is a major bottleneck in *Plasmodium* infection prompted us to investigate the mechanisms behind this inhibitory effect. In rodents, *Plasmodium* parasites take around 50–60 h to invade, develop and egress from the liver. Thus, we started by investigating the time frame of inhibition of *P*. *berghei* liver infection by *T*. *brucei*. Using a co-infection protocol similar to that described above, we assessed *Plasmodium* hepatic infection 30 min, 2 h, 6 h, 12 h, 24 h and 46 h after sporozoite injection. We observed that the *P*. *berghei* liver load of co-infected mice was ~55% lower than that of single-infected control mice as early as 30 minutes after sporozoite injection, and gradually decreased down to a ~95% reduction relative to control mice at 46 h post-infection (hpi) (**Fig 3A**).

A decrease in *Plasmodium* liver load may result from either a reduction in the number of infected hepatocytes or from a defect in the parasite’s capacity to replicate. To evaluate these two hypotheses, liver sections from single- or co-infected mice were analyzed by immunofluorescence microscopy at various time points of *Plasmodium* liver infection. We observed that the number of infected hepatocytes in co-infected mice was ~50% lower than in control mice at 6 hpi, and further decreased until 5% of that number at 46 hpi (**Fig 3B**), suggesting that most EEFs are eliminated during a co-infection. Of note, no significant differences were observed in the area of the *Plasmodium* EEF developing in the livers of control and co-infected mice at the selected time points (**Fig 3C and 3D**), indicating that an ongoing *T*. *brucei* infection does not inhibit the intrahepatic replication of the malaria parasite.

Collectively, our data show that the impairment caused by an ongoing *T*. *brucei* infection on a subsequent liver infection by *P*. *berghei* stems exclusively from a marked reduction in the number of *P*. *berghei*-infected hepatocytes, and is independent from the *P*. *berghei*’s ability to replicate inside these cells.

### Lymphocytes activated by *T*. *brucei* prevent hepatocyte invasion by *P*. *berghei*

**Fig 4:**
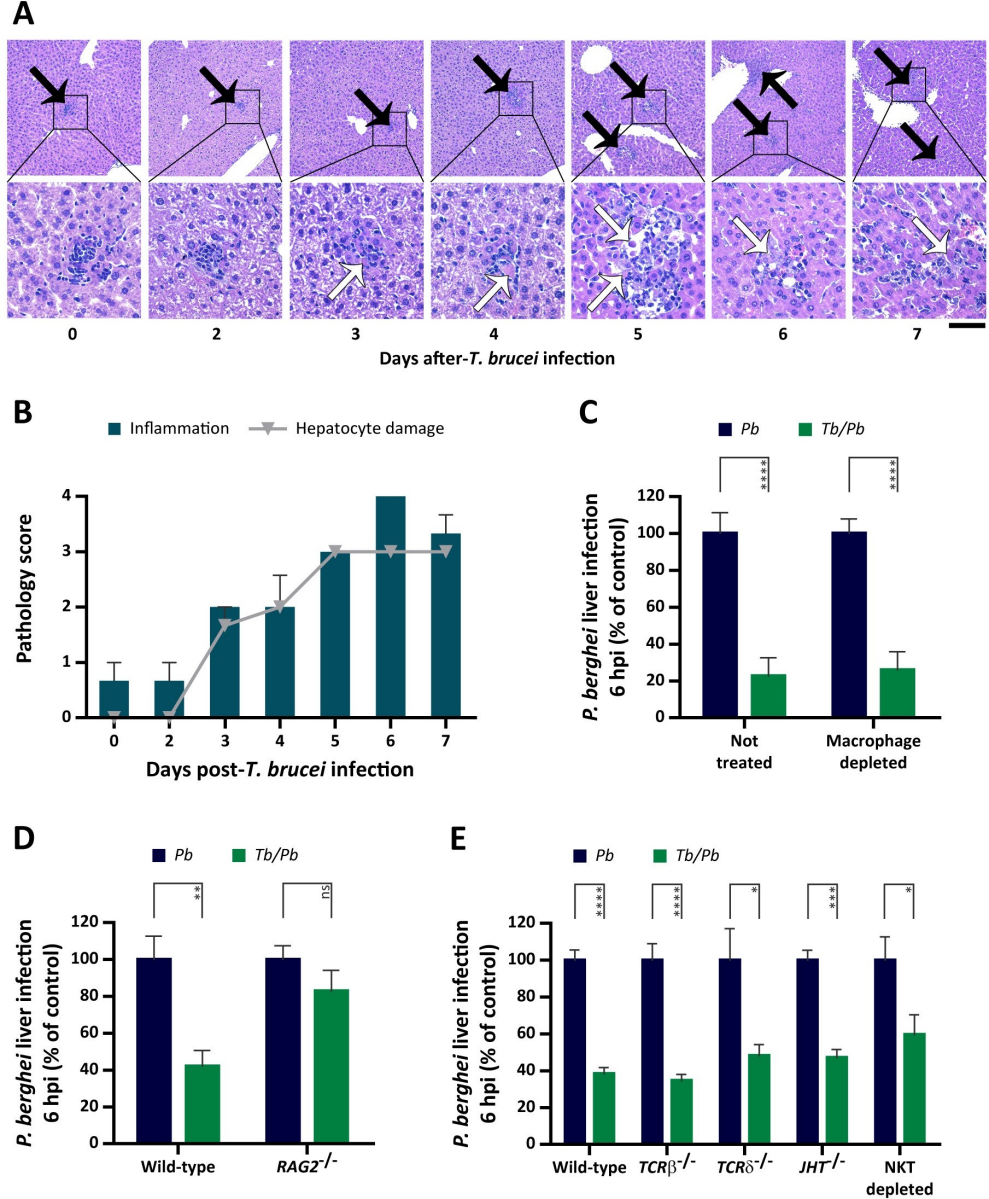
*T*. *brucei*-activated lymphocytes are required to inhibit *P*. *berghei* liver infection. **(A)** Representative microphotographs of liver from non-infected and *T. brucei*-infected mice (3–4 mice per time-point); depicted are the inflammatory cell infiltrates (black arrow) and hepatocellular damage/apoptosis seen at different time-points (XX axis) of *T*. *brucei* infection. Hematoxylin and Eosin. Original magnification 10x (upper panel; scale bar, 200 μm) and 40x (lower panel; scale bar, 50 μm). **(B)** Graphic representation of the severity of inflammatory cell infiltration (bars) and hepatocellular damage (dots), both scored through histopathology using a 5-tier system with 0–4 grading scale (0, absent; 1, minimal; 2, mild; 3, moderate; 4, marked). Time points indicated on the XX-axis correspond to the days of *T*. *brucei* infection. **(C)** *P*. *berghei* liver infection load quantified by qRT-PCR 6 h after injection of 3 x 10^4^ *P*. *berghei* sporozoites into naïve mice (*Pb*–blue bars) or mice infected 5 days earlier with *T*. *brucei* (*Tb/Pb*–green bars), non- or clodronate-treated 48 h prior to *P*. *berghei* infection. Bars represent the mean values of four independent experiments and error bars indicate the SEM. **(D)** *P*. *berghei* liver infection load quantification by qRT-PCR 6 h after injection of 3 x 10^4^ *P*. *berghei* sporozoites into wild-type and *RAG2*^-/-^ mice, either naïve (*Pb*–blue bars) or infected 5 days earlier with *T*. *brucei* (*Tb/Pb*–green bars). Bars represent the mean values of three independent experiments and error bars indicate the SEM. **(E)** *P*. *berghei* liver infection load quantification by qRT-PCR 6 h after injection of 3 x 10^4^ *P*. *berghei* sporozoites into wild-type, *TCRβ*^-/-^, *TCRδ*^-/-^, *JHT*^-/-^ and NKT depleted mice, either naïve (*Pb*–blue bars) or infected 5 days earlier with *T*. *brucei* (*Tb/Pb*–green bars). Bars represent the mean values of two to three independent experiments with error bars indicating the SEM. For **C** to **E**, the Mann-Whitney test was employed to assess the statistical significance of differences between the experimental groups. ns, not significant, * *P* < 0.05, *** *P* < 0.001 and **** *P* < 0.0001.

Given that the inhibitory effect of *T*. *brucei* on *Plasmodium* hepatic infection was only observed when *P*. *berghei* sporozoites were injected at least two days after trypanosome infection (**Fig 2B**), we hypothesized that this inhibitory effect could result from an immune response mounted against *T*. *brucei* infection. To test this hypothesis, we first assessed the liver immune landscape in *P*. *berghei*-only, *T*. *brucei*-only and co-infected mice by multi-parameter flow cytometry analysis of hepatic cells collected at a time point corresponding to 6 h after sporozoite injection. We found that whereas the relative proportions of liver immune cell subsets were not significantly different between the different groups of mice, the total number of leukocytes in the livers of *T*. *brucei*- and co-infected mice was increased relative to *P*. *berghei*-only or non-infected mice (**S6 Fig**). Microscopy analysis of histology liver sections revealed that, as early as three days after inoculation of *T*. *brucei*, there was minimal tissue damage, with apoptosis of few hepatocytes, and discrete foci of neutrophil infiltration (**Fig 4A and 4B**). At day 5 of *T*. *brucei* infection, hepatocellular apoptosis was marked and associated with multifocal infiltration by a population of mononuclear cells (**Fig 4A and 4B**), mostly composed of macrophages and lymphocytes (**Fig 4A**).

Since macrophages are able to eliminate sporozoites [34], we tested whether macrophages recruited to the liver during a *T*. *brucei* infection could be responsible for eliminating *Plasmodium* in this co-infection model. To this end, phagocytic cells were depleted from both naïve and *T*. *brucei*-infected mice by administration of clodronate-filled liposomes 48 h prior to sporozoite injection. The efficiency of phagocytic cell depletion was confirmed by quantification of mRNA levels of specific macrophage marker genes (Clec4f, F4/80 and CD68) in the liver [35], which showed a significant reduction in the number of macrophages in clodronate-treated mice (**S7A–S7C Fig**). We observed that phagocytic cell-depleted co-infected mice displayed a reduced *P*. *berghei* liver load, similar to that observed in non-treated co-infected mice (**Figs 4C** and **S8**), indicating that the impairment of *P*. *berghei* liver infection at 6 hpi by a primary *T*. *brucei* infection is independent of macrophages.

To assess the involvement of lymphocytes on the reduced ability of *P*. *berghei* sporozoites to establish a liver infection in the presence of *T*. *brucei*, mice genetically deficient for T and B cells (*RAG2*^*-/-*^) were co-infected and *Plasmodium* liver load was assessed 6 h after sporozoite injection. We observed that, in contrast to wild-type co-infected mice, which displayed a ~50% reduction in *P*. *berghei* liver load at this time point, *RAG2*^*-/-*^ co-infected mice displayed a *P*. *berghei* liver burden similar to that observed in *RAG2*^*-/-*^ single-infected mice (**Figs 4D** and **S8**). These results show that lymphocytes activated during a *T*. *brucei* infection inhibit a subsequent hepatocyte infection by *P*. *berghei* sporozoites. To identify the lymphocyte sub-population responsible for the observed phenotype, co-infections were performed in three distinct knock-out mouse strains, which lack either B, αβ or γδ T cells (*JHT*^*-/-*^, *TCRβ*^*-/-*^ and *TCRδ*^*-/-*^, respectively), as well as in mice depleted of NKT cells by injection of the anti-NK1.1 antibody. The efficiency of NKT cell depletion was confirmed by flow cytometry analysis (**S7D** and **S7E Fig**). We observed that, in the absence of each of these cell types, an ongoing *T*. *brucei* infection still impaired a subsequent *Plasmodium* infection (**Figs 4E** and **S8**), indicating that none of these subsets of cells is, on its own, responsible for the phenotype under investigation.

Overall, our data show that a *T*. *brucei* infection results in a strong inflammatory response in the liver that includes mononucleated immune cells. Although lymphocytes are necessary for *T*. *brucei*’s inhibitory effect, our results suggest that different lymphocyte subsets may synergistically mediate the observed impairment of *P*. *berghei* liver infection.

### Interferon-γ levels correlate positively with the impairment of *P*. *berghei* liver infection

**Fig 5:**
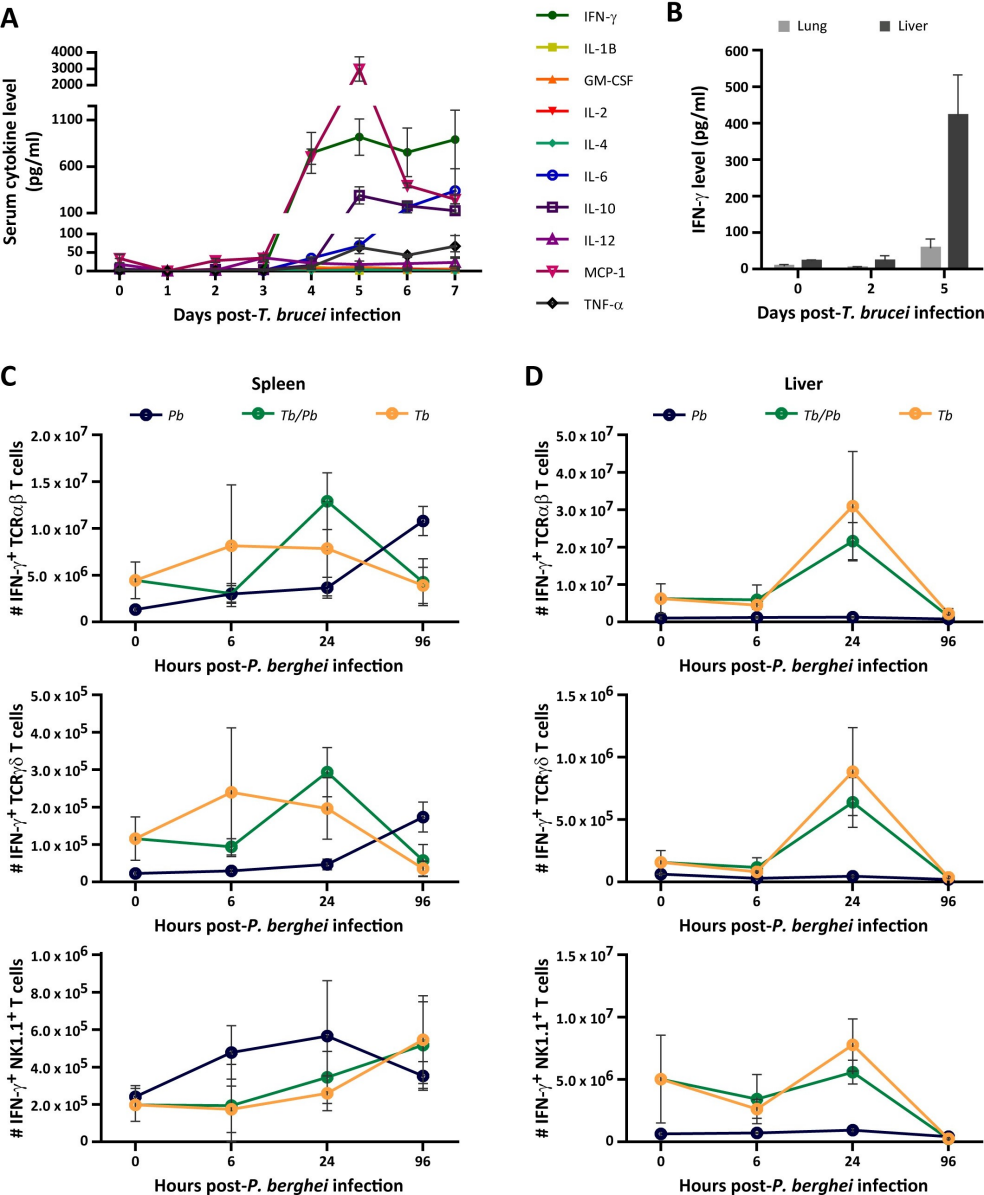
*T*. *brucei* elicits a strong immune response systemically and in the liver. **(A)** IFN-γ, IL-1B, GM-CSF, IL-2, IL-4, IL-6, IL-10, IL-12p70, MCP-1 and TNF-α quantification in serum by immunoassay from mice non-infected or infected only with *T*. *brucei*. The time points indicated on the XX-axis correspond to the days of *T*. *brucei* infection. Dots represent the mean values of three to four mice from one independent experiment with error bars indicating the SEM. **(B)** Quantification of IFN-γ by immunoassay in the lung (light grey bar) and liver (dark grey bar) of both non-infected mice and mice infected for two and five days with *T*. *brucei*. The time points indicated on the XX-axis correspond to the days of *T*. *brucei* infection. Bars represent the mean values of three to four mice from one independent experiment and error bars indicate the SEM. **(C-D)** Multi-parameter flow cytometry-based quantification of IFN-γ-producing TCRαβ, TCRγδ and NK1.1^+^ lymphocytes in the spleens (**C**) and livers (**D**) of mice infected only with *P*. *berghei* (*Pb*—blue dots), infected with *P*. *berghei* on the fifth day of *T*. *brucei* infection (*Tb/Pb–*green dots) and infected for 5 days only with *T*. *brucei* (*Tb*—yellow dots). The time points indicated on the XX-axis correspond to the time after injection of 3 x 10^4^ *P*. *berghei* sporozoites, with 0 h corresponding to day 5 of *T*. *brucei* infection. Dots represent the mean values of four mice from one independent experiment with error bars indicating SD.

Having demonstrated that the impairment of *P*. *berghei* liver infection by *T*. *brucei* is mediated by lymphocytes, and that this effect does not depend on a single lymphocyte population, we hypothesized that it could be dependent on the release of a specific factor produced by more than one lymphocyte subset. To investigate this, we first determined the serum levels of a panel of cytokines/chemokines throughout the first week of *T*. *brucei* infection. Our results showed that from the fourth day of *T*. *brucei* infection onwards, mice displayed an increase in the levels of IFN-γ, IL-1β, IL-2, IL-6, MCP-1 and TNF-α (**Fig 5A**). MCP-1, a chemokine that promotes migration and infiltration of monocytes/macrophages [36], displayed the highest fold-change increase at day 5 of *T*. *brucei* infection. However, given that phagocytic cells are not involved in the inhibition of *Plasmodium* liver infection by *T*. *brucei* (**Fig 4C**), we suspect that MCP-1 may not be crucial for mediating this impairment.

The second most abundant cytokine in the serum was IFN-γ, which is in agreement with previous reports [37, 38]. Next, we assessed IFN-γ levels in the liver, where the impairment of *P*. *berghei* infection is observed, and in the lungs, as a negative control. Our results showed that IFN-γ levels were 18-fold higher in the livers of *T*. *brucei*-infected mice than in those of control naïve mice, in contrast with the lung, where IFN-γ levels were similar in *T*. *brucei*-infected and in control mice (**Fig 5B**).

To confirm the importance of IFN-γ on the observed phenotype, we assessed the extent of protection against *P*. *berghei* hepatic infection and the corresponding levels of IFN-γ mRNA in the liver, following drug-mediated elimination of trypanosomes. To this end, mice infected with *T*. *brucei* for 4 days were treated with berenil and infected with *P*. *berghei* sporozoites 1 or 4 days post-treatment. We observed that the inhibitory effect of *T*. *brucei* on *P*. *berghei* hepatic infection 6 hpi is maintained when sporozoites are injected 1 day after the drug treatment but is significantly decreased when *P*. *berghei* is inoculated 4 days after berenil administration (**S9A Fig**), indicating that the protective effect of trypanosomes is progressively lost as they are eliminated from circulation. Crucially, the levels of IFN-γ mRNA in the livers of these mice correlated positively with the extent of protection against *P*. *berghei* hepatic infection by *T*. *brucei* (**S9B Fig**). Additionally, we quantified the levels of IFN-γ mRNA in the livers of *RAG2*^*-/-*^, as well as of *TCRβ*^*-/-*^, *TCRδ*^*-/-*^, *JHT*^*-/-*^ or NKT cell-depleted co-infected mice. Our results showed that in co-infected mice lacking individual subsets of lymphocytes, the levels of IFN-γ transcripts were similar to those detected in co-infected wild-type mice (**S10 Fig**), consistent with the fact that, in these mice, the inhibitory effect of *T*. *brucei* on *P*. *berghei* liver infection was still observed (**Fig 4E**). In contrast, IFN-γ levels were ~7-fold lower in *RAG2*^*-/-*^ than in wild-type co-infected mice (**S10 Fig**). Collectively, these results support a pivotal role for this cytokine in the phenotype under study.

We then sought to identify the systemic and hepatic cellular sources of IFN-γ. We quantified the number of IFN-γ-producing cells in spleen, as a proxy for systemic responses, and in the liver, at different times of infection of *P*. *berghei*-only, *T*. *brucei*-only and co-infected mice. We observed that IFN-γ production during the liver stage of *P*. *berghei* infection is substantially enhanced in all cell subsets from both organs, except for splenic NK1.1^+^ lymphocytes, from *T*. *brucei*- and co-infected mice relative to *P*. *berghei*-only or non-infected mice (**Fig 5C** and **5D**). These observations confirm that *T*. *brucei* infection leads to the production of IFN-γ by several subsets of lymphocytes, and that IFN-γ production is not abrogated by the individual absence of any of these cell populations (**S10A Fig**).

### Interferon-γ produced during *T*. *brucei* infection inhibits hepatocyte invasion by *P*. *berghei*

**Fig 6:**
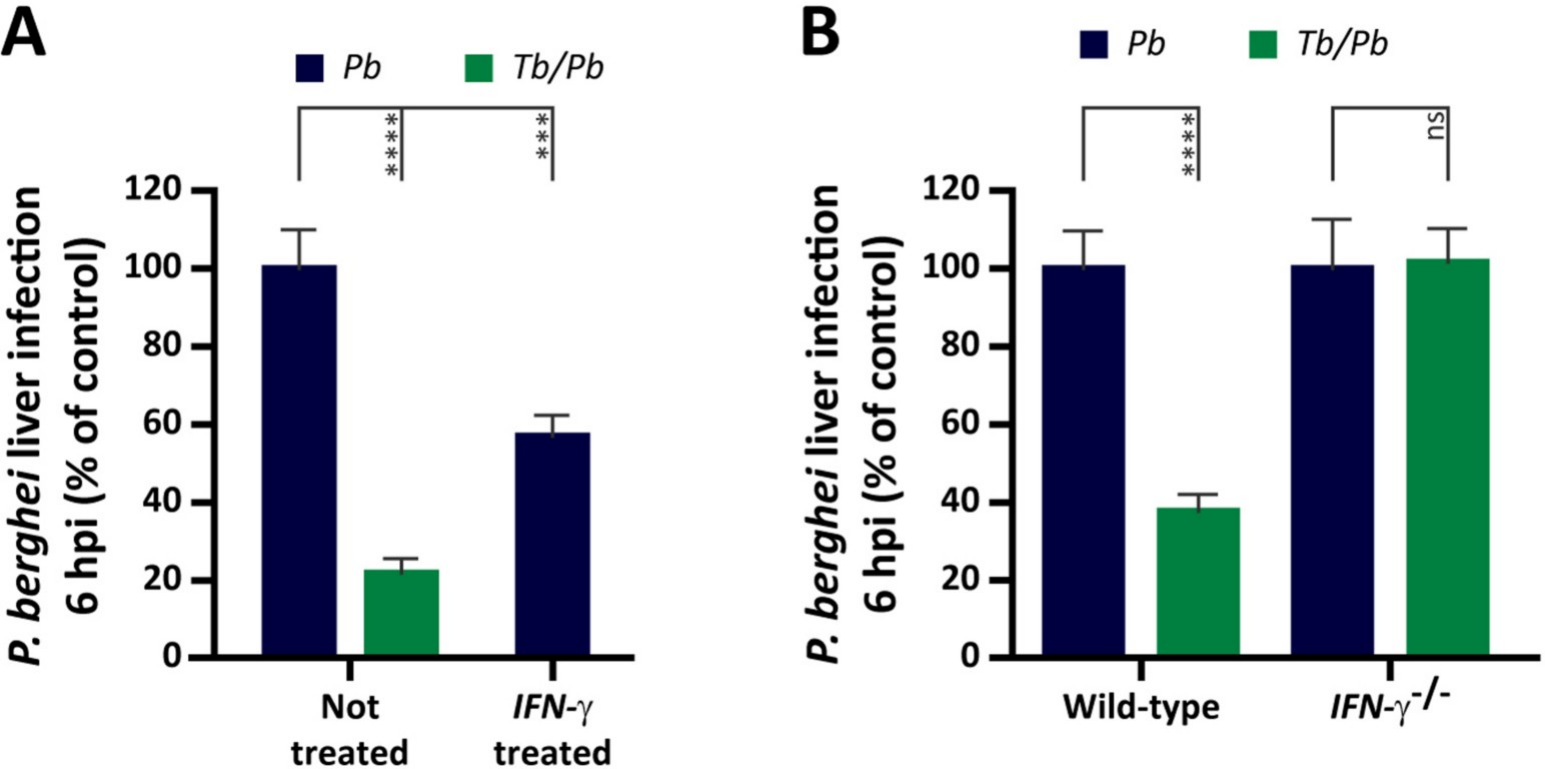
IFN-γ mediates the inhibitory effect of *T*. *brucei* on *P*. *berghei* liver infection. **(A)** *P*. *berghei* liver infection load quantification by qRT-PCR 6 h after injection of 3 x 10^4^ *P*. *berghei* sporozoites into naïve mice (blue bar), IFN-γ-treated mice (blue bar), or mice infected 5 days earlier with *T*. *brucei* (green bar). Bars represent the mean values of four independent experiments and error bars indicate the SEM. The one-way ANOVA with post-test Dunnett was employed to assess the statistical significance of differences between experimental groups. *** *P* < 0.001 and **** *P* < 0.0001. **(B)** *P*. *berghei* liver infection load quantification by qRT-PCR 6 h after injection of 3 x 10^4^ *P*. *berghei* sporozoites into wild-type and *IFN-γ*^-/-^ mice, either naïve (*Pb*–blue bars) or infected 5 days earlier with *T*. *brucei* (*Tb/Pb*–green bars). Bars represent the mean values of three independent experiments and error bars indicate the SEM. The Mann-Whitney test was employed to assess the statistical significance of differences between experimental groups. ns, not significant and **** *P* < 0.0001.

Mice treated with IFN-γ prior to *Plasmodium* sporozoite injection display a reduction in the ensuing liver infection [22–24, 39]. Since these studies have only assessed *Plasmodium* liver burden at 46 hpi, it remains unknown which stage of the *Plasmodium* liver infection was inhibited by IFN-γ. Thus, we wondered whether mice treated with IFN-γ would display a reduction in *P*. *berghei* hepatic load as early as 6 hpi. It has been described that only 0.2% of the IFN-γ administered i.p. reaches the serum, and that its half-life is ~2.16 h [40]. Therefore, to obtain IFN-γ serum levels in naïve mice equivalent to those present in the serum of mice infected with *T*. *brucei* for 5 days, 0.5 μg of recombinant IFN-γ was injected i.p. into mice 2 h prior to sporozoite injection, at the time of infection, and 2 h later. qRT-PCR analysis of liver samples collected 6 h after sporozoite injection revealed a reduction in the liver parasite load of mice pre-treated with IFN-γ similar to that observed in co-infected mice (**Fig 6A**).

To assess the function of IFN-γ in the *T*. *brucei*-mediated impairment of hepatocyte invasion by *P*. *berghei*, IFN-γ deficient mice (*IFN-γ*^*-/-*^) were subjected to the co-infection protocol described above. We found that, in contrast to wild-type mice, but similarly to *RAG2*^*-/-*^ mice, the *P*. *berghei* liver load of *IFN-γ*^*-/-*^ co-infected mice was comparable to that of single-infected *IFN-γ*^*-/-*^ mice (**Figs 6B** and **S8**), indicating that IFN-γ is necessary for the phenotype under study. Importantly, administration of recombinant IFN-γ to *RAG2*^*-/-*^ mice led to a partial reduction of *Plasmodium* liver load (**S10B Fig**), although less pronounced than that observed for wild-type mice (**Fig 6A**). This difference in magnitude is not surprising, as it is known that lymphocytes may respond to IFN-γ by further producing this cytokine [41], an effect that is absent in *RAG2*^*-/-*^ mice, which lack T and B lymphocytes.

Collectively, our data show that a primary *T*. *brucei* infection leads to the production of an array of pro-inflammatory cytokines/chemokines, among which lymphocyte-derived IFN-γ. We conclude that IFN-γ produced during a *T*. *brucei* infection is required for the impairment of a subsequent hepatocyte infection by *P*. *berghei*. Importantly, our results show for the first time that IFN-γ exerts a marked inhibitory effect during the very early stages (up to 6 hpi) of *Plasmodium* liver infection.

## Discussion

Several studies have demonstrated that the course of a *Plasmodium* infection can be influenced by the presence of a second pathogen in the same host, either by aggravating malaria-associated pathology [21, 42], or by conferring protection against disease severity [43, 44]. However, despite the geographical overlap between the etiological agents of sleeping sickness and malaria, whether an ongoing infection by *T*. *brucei* has an impact on a subsequent infection by *Plasmodium* had hitherto not been assessed. We hypothesized that the strong immune response elicited by a *T*. *brucei* infection [45] could limit the establishment and/or development of a subsequent infection by the malaria parasite. Our results showed that mice with a patent *T*. *brucei* infection, and detectable parasitemia, and subsequently infected with *P*. *berghei* sporozoites, display a reduction in the ensuing liver infection by the malaria parasite, relative to mice infected only with *P*. *berghei*. We further showed that the impairment of *P*. *berghei* liver infection is observed from very early stages of hepatic infection by the malaria parasite, and that the number of *Plasmodium* intrahepatic forms in co-infected mice continuously decreases until the end of the liver stage of infection.

Liver damage is a frequent sign of pathology during African Trypanosomiasis [46–49], and mononuclear cell infiltrates have been observed in the livers of *T*. *brucei*-infected animals, at a late stage of infection [47, 50, 51]. In fact, liver mononuclear immune cells, including resident Kupffer cells, have been suggested to play a critical role in trypanosome clearance from the bloodstream [52–54]. The histopathology analysis of liver sections collected from mice infected only by *T*. *brucei* revealed that hepatocyte damage can be observed as early as 3 days after inoculation with trypanosomes, and might result from the infiltration and multifocal distribution of inflammatory cells [55]. Of note, when sporozoites were administered to mice on the second day of *T*. *brucei* infection, a time when the livers of those mice were still undamaged, *P*. *berghei* liver infection in co-infected mice was similar to that observed in *P*. *berghei* single-infected mice. Conversely, we found that mice infected for 5 days with *T*. *brucei*, corresponding to the day of *P*. *berghei* sporozoite injection in our studies, displayed significant liver inflammation and hepatocellular damage. Therefore, we hypothesized that the hyperinflammatory state of mice infected with *T*. *brucei* could be responsible for the inability of *P*. *berghei* sporozoites to establish a liver infection. We showed that *T*. *brucei-*activated lymphocytes are required to impair a subsequent *P*. *berghei* infection, and showed that this impairment is mediated by IFN-γ. During a *T*. *brucei* infection, liver NK, NKT and CD8^+^ T cells are the early sources of IFN-γ, which is required to further recruit neutrophils and monocytes that are responsible for the direct elimination of trypanosomes [38, 54, 56]. Interestingly, it has also been shown that a primary infection by *P*. *yoelii* triggers a type-I interferon signaling cascade, leading to an enrichment of NKT cells in the liver, which produce IFN-γ and inhibit a subsequent infection by the same parasite [57]. We found that the levels of IFN-γ mRNA in the livers of *T*. *brucei*-infected mice lacking T and B cells were significantly lower than those observed in wild-type control mice. Interestingly, IFN-γ and IL-4 have been implicated in the protection conferred by *Schistosoma* against a subsequent *P*. *yoelii* hepatic infection [58]. Although *T*. *brucei* does not promote the production of IL-4, the load of IFN-γ appears to be sufficient to protect against a *Plasmodium* infection.

Treatment with IFN-γ, both *in vitro* and *in vivo*, leads to a reduction in the number of *P*. *berghei* intrahepatic forms present at the late stages of *P*. *berghei* liver infection [22–24, 39]. Our results reveal for the first time that IFN-γ reduces the number of *P*. *berghei* intrahepatic forms as early as 6 hours post-sporozoite injection. Both a general increase in the number of leukocytes recruited to the liver and an overall enhancement in IFN-γ production are observed in *T*. *brucei*-only- and co-infected mice, indicating that, during the co-infection, the immune response is mainly dictated by the primary infection. Since IFN-γ levels throughout infection by *T*. *brucei* have been shown to closely mirror its parasitemia curve [59], the impairment in *P*. *berghei* infection shown in **Fig 2B** follows a similar trend to those of both *T*. *brucei* parasitemia and IFN-γ serum levels. Additionally, the progressively smaller inhibitory effect of trypanosomes as they are eliminated from circulation by drug treatment is accompanied by a decrease in the levels of IFN-γ mRNA in the liver, which further supports the conclusion that IFN-γ is a critical factor in the *T*. *brucei*-mediated impairment of *P*. *berghei* liver infection. Also of note, co-infected mice display a marked increase in the number of hepatic cells capable of producing IFN-γ at 24 h after *P*. *berghei* sporozoite injection (**Fig 5D**), which may contribute to explaining the progressive decrease observed in the number of EEFs from 6 to 46 hpi (**Fig 3B**).

Despite IFN-γ‘s implication in the recruitment of macrophages to the liver and in their activation during the early phases of a *T*. *brucei* infection [54, 60–62], and the capacity of macrophages to eliminate sporozoites upon the establishment of a *P*. *berghei* liver infection [34], our results do not suggest an involvement of these cells in the impairment of *P*. *berghei* liver infection by *T*. *brucei*. Since the IFN-γ-mediated elimination of *Plasmodium* intrahepatic forms has been shown to either involve nitric oxide production [23] or the induction of noncanonical autophagy pathway [63], we propose that at least one of these processes may dictate the impairment of *P*. *berghei* hepatic infection by *T*. *brucei*. Further investigation is required to fully clarify the mechanism whereby IFN-γ mediates the impairment of the establishment of *P*. *berghei* hepatic infection in the context of a co-infection with *T*. *brucei*.

In a *S*. *mansoni*/*P*. *chabaudi* co-infection model, increased levels of IFN-γ render A/J co-infected mice less susceptible to infection by the malaria parasite, relative to that observed in schistosome-free A/J mice [64]. In contrast, mice co-infected with *T*. *crassiceps* and *P*. *yoelli* survived longer than mice infected with the malaria parasite only, which the authors correlated with a reduction in IFN-γ levels [65]. These studies point to a crucial role for IFN-γ in modulating a secondary *Plasmodium* infection. Interestingly, in mice infected only with *Plasmodium*, divergent roles for IFN-γ on the development of severe malaria have been described (reviewed in [66]). Whereas early production of IFN-γ has been shown to protect against ECM [67], IFN-γ production by CD4^+^ T cells can lead to CD8^+^ T cell accumulation in the brain and promotes the development of ECM [68]. We showed that *T*. *brucei*-mediated protection against erythrocytic *Plasmodium* infection and ECM is not time-restricted and still occurs when *P*. *berghei* sporozoites are injected 15 days after the primary infection, a time when *T*. *brucei* parasitemia is detectable, and serum IFN-γ levels are high [59]. Our data further reveal an early peak in the number of hepatocytes that are able to produce IFN-γ at 24 h after *P*. *berghei* sporozoite injection in co-infected mice (**Fig 5D**), consistent with that reported to confer protection against ECM [67]. Collectively, these observations suggest that, besides its inhibitory effect on *P*. *berghei* hepatic infection, IFN-γ may also have an impact on malaria-associated pathology.

During a *T*. *cruzi* infection, NK cells are the major source of IFN-γ which is required to limit parasite replication [69]. Interestingly, mice co-infected with *T*. *cruzi* and *P*. *berghei* do not develop ECM symptoms, presumably due to a reduction in the accumulation of CD8^+^ T cells in the brain of those animals [44]. Therefore, during a *T*. *brucei*/*P*. *berghei* co-infection, the accumulation of IFN-γ by *T*. *brucei*-activated lymphocytes prior to the onset of *Plasmodium* parasitemia may limit the accumulation of CD8^+^ T cells and reduce malaria severity in the co-infected mice.

Our results showed that the reduction of *P*. *berghei* hepatic infection by *T*. *brucei* prevented the appearance of *Plasmodium* parasitemia in 50% and in 100% of C57Bl/6J and BALB/cByJ co-infected mice, respectively. We further observed that co-infected mice that developed *P*. *berghei* parasitemia did not display symptoms of severe malaria and survived significantly longer than mice that had not been exposed to trypanosomes. In order to assess the impact of trypanosomiasis on the blood stage of *Plasmodium* infection, the liver stage of the malaria parasite's life cycle was bypassed by inoculating *P*. *berghei*-infected RBCs into *T*. *brucei*-infected or control mice. Although *P*. *berghei* parasitemia became detectable in all the mice with an ongoing *T*. *brucei* infection, co-infected mice never presented severe malaria symptoms and survived longer than mice that had not been infected with trypanosomes prior to *P*. *berghei* inoculation. Collectively, our results show for the first time that a primary *T*. *brucei* infection renders mice more resistant to malaria, which happens as a consequence not only of a *T*. *brucei*-dependent reduction in the *P*. *berghei* hepatic infection, but also of a direct effect of the *T*. *brucei* infection on a *P*. *berghei* blood infection.

To the best of our knowledge, this is the first report addressing whether the outcome of an infection with the malaria parasite is affected by an ongoing *T*. *brucei* infection. Our results show that a primary *T*. *brucei* infection leads to the production of an array of pro-inflammatory cytokines/chemokines, among which lymphocyte-derived IFN-γ, which significantly reduces a subsequent hepatic infection by *P*. *berghei* sporozoites. Since IFN-γ and T cells have been extensively implicated in vaccine-mediated protection against *Plasmodium* liver infection (reviewed in [70–72]), it would be interesting to assess the impact of an ongoing infection by a pathogen that elicits a high IFN-γ response, such as *T*. *brucei*, on the efficiency of malaria vaccination. Moreover, the ensuing *P*. *berghei* blood infection was absent in most of the co-infected mice and for those in which the malaria parasite could establish a blood infection, the co-infected mice never presented severe symptoms and survived longer. In view of the geographical overlap between infections by *T*. *brucei* and *Plasmodium*, it would be interesting to assess whether individuals suffering from sleeping sickness in these regions display some degree of protection against severe malaria.

## Materials and methods

### Mice

Male C57BL/6J and BALB/cByJ mice (6–8 weeks old) were purchased from Charles River Laboratories (Lyon, France). *RAG2*^-/-^, *TCRβ*^-/-^ and *JHT*^-/-^ mice (7–10 weeks old) were bred and obtained from the specific pathogen-free (SPF) facilities at Instituto Gulbenkian de Ciência (Oeiras, Portugal). *TCRδ*^-/-^ and *IFN-γ*^-/-^ mice (7–10 weeks old), kindly provided by Bruno Silva-Santos, were bred in SPF facilities at Instituto de Medicina Molecular João Lobo Antunes (iMM JLA) (Lisbon, Portugal). All the animals were housed and kept in the SPF rodent facility of the iMM JLA, a licensed establishment that complies with Directive 2010/63/EU.

### Ethics statement

All the experimental animal work was performed in strict compliance to the guidelines of our institution’s animal ethics committee, who also approved this study (under authorization AWB_2015_09_MP_Malaria), and in accordance with the Federation of European Laboratory Animal Science Associations (FELASA) guidelines.

### Parasites and infections

The transgenic 90–13 cell-line of the *Trypanosoma brucei brucei* AnTaT 1.1 pleomorphic strain [73] (hereafter referred to as *T*. *brucei*) was employed throughout this study. Mice were infected by intraperitoneal (i.p.) injection of 2 x 10^3^ motile parasites, obtained from cryostabilates, as previously described [8, 50]. GFP [74]- or luciferase [75]-expressing *Plasmodium berghei* ANKA rodent malaria parasites (hereafter referred to as *P*. *berghei*) and *Plasmodium yoelii yoelii* 17XNL [76] (hereafter referred to as *P*. *yoelii*) were used throughout this study. For the assessment of mosquito feeding preferences, 6–10 *P*. *berghei*-infected mosquitoes were allowed to feed on uninfected or *T*. *brucei*-infected mice for 30 min in the dark, and the percentage of mosquitoes that ingested a blood meal was determined by visual inspection of their midguts. For intravenous (i.v.) sporozoite injections, *P*. *berghei* and *P*. *yoelii* sporozoites were obtained by dissection of salivary glands from infected female *Anopheles stephensi* mosquitoes, reared at iMM JLA. Mice were infected by retro-orbital i.v. injection of 500 or 3 x 10^4^ sporozoites, under isoflurane anesthesia. *P*. *berghei*-infected RBCs (iRBCs) were obtained from an infected homologous donor mouse. Mice were infected by i.p. injection of 1 x 10^6^ iRBCs.

### Assessment of experimental cerebral malaria (ECM) symptoms

ECM development was monitored daily using the rapid murine coma and behavior scale (RMCBS) score, as described in [77]. Mice with RMCBS score equal or below 5/20 were classified as displaying ECM symptoms and euthanized immediately.

### Assessment of parasitemia

*T*. *brucei* parasitemia was assessed by counting the number of trypanosomes in blood samples, collected from the tail vein, using an Olympus CKX31 inverted microscope (Olympus, Tokyo, Japan). *P*. *berghei* parasitemia was monitored daily by flow cytometry, using a drop of tail-blood diluted in phosphate-buffered saline (PBS), as previously described [78]. GFP-expressing parasites were detected in the green fluorescent channel, FL1-H, using a BD LSRFortessa X-20 (BD Biosciences, Franklin Lakes, NJ, USA) flow cytometer with the FACSDiva software (version 8.0, BD BIOSCIENCES, Franklin Lakes, NJ, USA). The collected data was after analyzed with FlowJo software (version 10.0, Ashland, OR, USA) and results are expressed as the percentage of iRBCs in the sample.

### Quantification of *in vivo P. berghei* hepatic infection

Mouse hepatic infection was assessed at either 30 min, 2 h, 6 h, 12 h, 24 h or 46 h after *P*. *berghei* sporozoite inoculation and quantified either by real-time *in vivo* imaging employing the IVIS Lumina Imaging System (Caliper LifeSciences, Waltham, MA, USA), as previously described [75], or by quantitative real-time reverse transcriptase-PCR (qRT-PCR), also as previously described [79]. *P*. *berghei* bioluminescence was measured as total flux (photons/s) and analyzed with the Living Image software (version 3.0, PerkinElmer, Waltham, MA, USA). For qRT-PCR analyses, 0.7–0.9 mg of livers collected by dissection of infected mice were mechanically homogenized in TRIzol (BioRad, Hercules, CA, USA), RNA was extracted following the manufacturer’s instructions and converted into complementary DNA (cDNA) as described below. Liver *P*. *berghei* load was quantified by qRT-PCR, as previously described [80], using primers specific for *P*. *berghei* 18S rNA (Table 1). Expression of the endogenous mouse housekeeping gene hypoxanthine-guanine phosphoribosyltransferase (*Hprt*) was used for normalization.

**Table 1:** List of primer sequences.

Target gene	Forward primer (5’-3’)	Reverse primer (5’-3’)
*P. berghei 18S rRNA*	AAGCATTAAATAAAGCGAATACATCCTTAC	GGAGATTGGTTTTGACGTTTATGTG
*CLEC4f*	TGAGTGGAATAAAGAGCCTCCC	TCATAGTCCCTAAGCCTCTGGA
*CD68*	AGCTGCCTGACAAGGGACACT	AGGAGGACCAGGCCAATGAT
*F4/80*	CCCAGCTTATGCCACCTGCA	TCCAGGCCCTGGAACATTGG
*IFN-γ*	CACACTGCATCTTGGCTTTG	TCTGGCTCTGCAGGATTTTC
*Hprt*	TTTGCTGACCTGCTG GATTAC	CAAGACATTCTTTCCAGTTAAAGTTG

### RNA extraction, complementary DNA synthesis and qRT-PCR

RNA was extracted following the manufacturer’s instructions and quantified using a NanoDrop DR 1000 Spectrophotometer (Thermo Fisher Scientific, Waltham, MA USA). cDNA was synthesized from 1 μg of RNA, using the NZYTech cDNA synthesis kit (NZYTech, Lisbon, Portugal), according to the manufacturer’s recommendations, and employing the following thermocycling parameters: 25°C for 10 min, 55°C for 30 min, and 85°C for 5 min. The qRT-PCR reaction was performed in a total volume of 10 μl reaction employing an Applied Biosystems StepOne Plus equipment (Applied Biosystems, Foster City, CA, USA), using the SYBR Green PCR Master Mix (Applied Biosystems) and the following thermocycling parameters: 50°C for 2 min, 95°C for 10 min, 40 cycles at 95°C for 15 s and 60°C for 1 min, melting stage was done at 95°C for 15 s, 60°C for 1 min, and 95°C for 30 s. Primer pairs used to detect target gene transcripts are listed in Table 1. Gene expression was analyzed by the comparative CT method (ΔΔCT) and the expression level of all target genes was normalized to that of *Hprt*.

### Immunofluorescence microscopy analysis of *in vivo P. berghei* hepatic infection

Livers were collected at either 6, 12, 24 or 46 h after *P*. *berghei* sporozoite inoculation, and fixed in 4% (v/v) paraformaldehyde (PFA) (SantaCruz Biotechnology, Dallas, TX, USA) for at least 12 h at room temperature (RT). Liver sections of 50 μm thickness were stained and analyzed as previously described [79, 81]. Briefly, slices were incubated in permeabilization/blocking solution containing 1% (w/v) bovine serum albumin (Sigma-Aldrich, St. Louis, MO, USA) and 0.5% (v/v) Triton-X100 in PBS (Sigma-Aldrich) at RT for 1 h, followed by a 2 h incubation at RT with an anti-UIS4 antibody (goat, homemade; dilution 1:500). Liver sections were further incubated for 1 hour in a 1:500 dilution of anti-GFP-Alexa 488 antibody (Invitrogen, Carlsbad, CA, USA) and anti-goat Alexa-Fluor 568 (Invitrogen, Carlsbad, CA, USA) in the presence of a 1:1,000 dilution of Hoechst 33342 (Invitrogen, Carlsbad, CA, USA). After washing, liver sections were mounted on microscope slides with Fluoromount (SouthernBiotech, Birmingham, AL, USA). Widefield images for *P*. *berghei* intrahepatic forms’ size determination were acquired employing a Zeiss Axiovert 200M microscope (Carl Zeiss, Oberkochen, Germany). Confocal images were acquired using a Zeiss LSM 510 confocal microscope (Carl Zeiss, Oberkochen, Germany). Images were processed with the ImageJ software (version 1.47, NIH, Bethesda, MD, USA).

### Treatment of *T*. *brucei* infection

Trypanosomes were eliminated by i.p. injection of 250 ng of Berenil (Sigma-Aldrich), 4 days after *T*. *brucei* infection. *P*. *berghei* sporozoites were inoculated into treated mice 1 or 4 days after drug administration. Livers were collected 6 h after sporozoite injection and processed as described above for the quantification of *P*. *berghei* liver load by qRT-PCR.

### Liver histopathology

Livers collected from mice infected for 2 to 7 days with *T*. *brucei* or from non-infected mice used as controls were formalin-fixed in neutral buffered formalin, paraffin-embedded, cut in 4 μm sections, and stained with hematoxylin (Bio-Optica, Milan, Italy) and eosin (Thermo Fisher Scientific). Tissue sections were analyzed by a pathologist blinded to experimental groups in a Leica DM2000 microscope coupled to a Leica MC170 HD microscope camera (Leica Microsystems, Wetzlar, Germany). Inflammatory cell infiltration and hepatocellular damage were scored using a 5-tier system with 0–4 grading scale (0, absent; 1, minimal; 2, mild; 3, moderate; 4, marked).

### Isolation of liver and spleen leukocytes

Livers and spleens were collected at 0 h, 6 h, 24 h and 96 h after *P*. *berghei* sporozoite inoculation, mechanically homogenized in PBS containing 2 U/ml DNAse (Sigma-Aldrich), using a 100 μm cell strainer, and centrifuged at 410 x g for 8 min at room temperature (RT). Liver cell pellets were further fractionated using a 35% (v/v) Percoll gradient medium (Sigma-Aldrich) diluted in Roswell Park Memorial Institute 1640 medium (RPMI) (Gibco-Thermo Fisher Scientific, Waltham, MA USA), and centrifuged at 1,360 x g for 20 min without brake at 20°C. Liver and spleen cell pellets were washed with PBS and centrifuged at 410 x g for 8 min at RT. RBCs were depleted by a 3 min incubation at RT with ammonium-chloride-potassium solution, which was stopped by washing with PBS 2% FBS (Gibco-Thermo Fisher Scientific) (FACS buffer). The leukocyte suspension was centrifuged at 410 x g for 8 min at RT and resuspended in PBS for subsequent staining.

### Analysis of liver and spleen leukocytes by flow cytometry

One million leukocytes from each mouse were plated in 96-well plates, centrifuged at 845 x g for 3 min at 4°C and incubated with α-CD16/CD32 (eBioscience/Thermo Fisher Scientific, Waltham, MA, USA) for 20 min at 4°C. Liver and/or spleen leukocytes were there surface-stained for 20 min at 4°C using appropriate combinations of the following fluorochrome-conjugated anti-mouse monoclonal antibodies: FITC or eFluor450anti-TCRγδ (clone GL3), PE anti-NKp46 (clone 29A1.4), PerCP/Cy5.5 anti-CD3ɛ (clone 145-2C11), PE or PE/Cy7 anti-NK1.1 (clone PK136), Alexa Fluor 647 anti-SiglecF (clone ES0-2440), PE/Cy7 anti-Ly6G (clone AE8), FITC or APC/Cy7 anti-CD11b (clone M1/70), Brilliant Violet (BV)421 or BV510 anti-CD4 (clone RM4-5), BV510 or Alexa Fluor 700 anti-CD45 (clone 30-F11), BV605 anti-CD19 (clone 6D5), BV605 anti-Ly6C (clone HK1.4), and BV711 anti-CD8α (clone 53–6.7), plus eFluor 506 or eFluor 780 Fixable Viability Dye (eBioscience, Thermo Fisher Scientific) for dead cell exclusion. All antibodies were from eBioscience (Thermo Fisher Scientific) or Biolegend. Cells were acquired on a BD LSRFortessa or LSRFortessa X-20 (BD Biosciences) and data acquisition and analysis were carried out using the FACSDiva (version 6.2) and FlowJo (version 10.5.3, FlowJo) software packages, respectively.

### *In vivo* depletion of macrophages

Macrophages were depleted by i.v. injection of 1 mg of liposome-encapsulated clodronate (Liposoma B.V., Amsterdam, The Netherlands), 48 h prior to *P*. *berghei* sporozoite inoculation. An equivalent volume of liposome-encapsulated PBS was injected i.v. into control mice. Livers were collected 6 h after sporozoite injection and processed as described above for the quantification of *P*. *berghei* liver load by qRT-PCR.

### *In vivo* depletion of Natural Killer (NK) and Natural Killer T cells (NKT)

NK and NKT cells were depleted by i.v. injection of 200 μg of anti-NK1.1 (clone PK136, Bio X Cell, West Lebanon, NH, USA), 24 hours prior to *P*. *berghei* sporozoite inoculation. Livers were collected 6 h after sporozoite injection and processed as described above for the quantification of the *P*. *berghei* liver load by qRT-PCR.

### Quantification of serum, liver and lung cytokines/chemokines

Blood was collected by heart puncture of mice infected 2 to 7 days earlier with *T*. *brucei*. After clotting, samples were centrifuged at 1,000 x g for 10 min at 4°C, and serum was transferred to a fresh tube. Livers and lungs were obtained by dissecting mice infected 2 to 7 days earlier with *T*. *brucei* and homogenized in lysis buffer containing 20 mM Tris HCl, pH 7.5, 0.5% (v/v) Tween 20, 150 mM NaCl, and 1% (v/v) protease inhibitor cocktail in PBS. Tissue homogenates were centrifuged at 10,000 x g for 10 min at 4°C and the supernatants transferred to fresh tubes. Uninfected mice were used as controls. The serum levels of an array of pro-inflammatory cytokines/chemokines (IFN-γ, IL-1B, GM-CSF, IL-2, IL-4, IL-6, IL-10, IL-12p70, MCP-1 and TNF-α) were determined using the multiplexing addressable laser bead immunoassay (Eve Technologies, Calgary, Canada).

### Quantification of IFN-γ-producing lymphocytes in the liver and spleen by flow cytometry

One million liver or spleen leukocytes were stimulated in a 96-well plate for 4 h in the presence of 50 ng/ml PMA, 500 ng/mL ionomycin and 10 μg/ml brefeldin (all from Sigma). After this period, cells were Fc-blocked (using α-CD16/CD32) and surface-stained, as described above, using the following monoclonal antibodies: FITC anti-CD11b, eFluor450 anti-TCRγδ, Alexa Fluor 700 anti-CD45 and PE/Cy5.5 anti-NK1.1, plus eFluor 780 fixable viability dye for dead cells discrimination. Cells were then washed with FACS buffer, followed by fixation and permeabilization using the Transcription Factor Staining Buffer Set, according to the manufacturer’s instructions (eBioscience, Thermo Fisher Scientific). Intracellular staining was performed for 30 min at 4°C using BV510 anti-CD4, BV711 anti-CD8α, PE anti-IFN-γ (clone XMG1.2; BD Pharmingen) and PE/Cy7 anti-CD3ɛ. Cells were washed, resuspended in FACS buffer and acquired on a BD LSRFortessa X-20 (BD Biosciences). Data was analyzed using FlowJo v10.5.3 (FlowJo, LLC).

### *In vivo* treatment with IFN-γ

IFN-γ was administered to mice by i.p. injection of 0.5 μg of recombinant murine IFN-γ (PeproTech, Rocky Hill, NJ, USA), 2 hours prior to inoculation of *P*. *berghei* sporozoites, at the time of infection and 2 h later. Livers were collected 6 h after sporozoite injection and processed as described above for the quantification of the *P*. *berghei* liver load by qRT-PCR.

### Statistical analyses

Data are expressed as mean ± standard deviation (SD) or standard error of the mean (SEM). Statistical analyses were performed using the GraphPad Prism 6 software (La Jolla, CA, USA). Significant differences were determined using One-way analysis of variance (ANOVA), Log-Rank (Mantel-Cox) test, Two-way ANOVA or Two-tailed Mann-Whitney test. Significances are represented as indicated in each figure: ns–not significant, * *P* < 0.05, ** *P* < 0.01, *** *P* < 0.001 and **** *P* < 0.0001.

## Supporting information

S1 Fig *A. stephensi* mosquitoes display a feeding preference for *T. brucei*-infected relative to uninfected mice. Assessment of mosquito feeding on uninfected or *T*. *brucei*-infected BALB/cByJ **(A)** or C56BL/6J **(B)** mice. Percentage of mosquitoes that ingested a blood meal and SEM of the pooled data of 15 mice from three independent experiments for each mouse strain are shown. Each dot represents one mouse exposed to the bites of 6 (1 experiment per mouse strain) or 10 (2 experiments per mouse strain) *P*. *berghei*-infected mosquitoes.
(TIF)

S2 Fig *T. brucei* attenuates *P. berghei* infection in BALB/cByJ and C56BL/6J. **(A)** Assessment of *P*. *berghei* parasitemia by flow cytometry after inoculation of 500 sporozoites into naïve BALB/cByJ mice (*Pb*—blue line) or BALB/cByJ mice infected 5 days earlier with *T*. *brucei* (*Tb/Pb*—green line). Percentage of iRBCs and SEM of the pooled data of 10 mice from two independent experiments is shown. **(B)** Assessment of *P*. *berghei* parasitemia by flow cytometry after inoculation of 500 sporozoites into naïve C57BL/6J mice (*Pb*—blue line) or C57BL/6J mice infected 5 days earlier with *T*. *brucei* (*Tb/Pb*—green line). Percentage of iRBCs and SEM of the pooled data of 10 mice from two independent experiments are shown.
(TIF)

S3 Fig *P. berghei* infection does not affect *T. brucei* parasitemia. Optical microscopy-based assessment of *T*. *brucei* parasitemia in C57BL/6J mice infected only with trypanosomes (*Tb–*light green line) or inoculated with 1 x 10^6^ *P*. *berghei*-iRBCs 5 days after infection with *T*. *brucei* (*Tb/Pb–*dark green line). The geometrical means of the number of trypanosomes per ml of blood and SD of the pooled data of 5 mice from one experiment are shown.
(TIF)

S4 Fig *T. brucei* attenuates hepatic infection by *P. yoelii*. *P*. *yoelii* liver infection load determined by qRT-PCR 46 h after sporozoite injection into naïve mice (blue bar) or mice previously infected by *T*. *brucei* (green bar). Bars represent the mean values of two independent experiments and error bars indicate the SEM. The Mann-Whitney test was employed to assess the statistical significance of differences between experimental groups (*** *P* < 0.001).
(TIF)

S5 Fig *T. brucei*-mediated protection against *P. berghei* erythrocytic infection and ECM is not time-restricted. **(A)** Assessment of *P*. *berghei* prepatency period following inoculation of 3 x 10^4^ *P*. *berghei* sporozoites into naïve C57BL/6J mice (*Pb* - blue line) or C57BL/6J mice infected 15 days earlier with *T*. *brucei* (*Tb/Pb* - green line). Percentage of mice displaying *P*. *berghei* parasitemia, as measured by flow cytometry. The pooled data of 4–6 mice from one independent experiment is shown. **(B)** Assessment of *P*. *berghei* parasitemia by flow cytometry after inoculation of 3 x 10^4^ GFP-expressing *P*. *berghei* sporozoites into naïve C57BL/6J mice (*Pb–*blue line) or C57BL/6J mice infected 15 days earlier with *T*. *brucei* (*Tb/Pb–*green line). Percentage of iRBCs and SEM of the pooled data of 4–6 mice from one independent experiment are shown. **(C)** Mouse survival following inoculation of 3 x 10^4^ *P*. *berghei* sporozoites into naïve C57BL/6J mice (*Pb–*blue line) or C57BL/6J mice infected 15 days earlier with *T*. *brucei* (*Tb/Pb–*green line). The pooled data of 4–6 mice from one independent experiment is shown. Time window for ECM development is depicted by the grey-shaded area.
(TIF)

S6 Fig *T. brucei* infection leads to a recruitment of leukocytes to the liver. Multi-parameter flow cytometry-based quantification of leukocytes in the liver of mice not infected, infected for 5 days with *T*. *brucei* (*Tb*), co-infected with *P*. *berghei* and *T*. *brucei* (*Tb*/*Pb*), or infected only with *P*. *berghei* (*Pb*). Livers were collected 6 h after injection of 3 x 10^4^ *P*. *berghei* sporozoites into naïve mice (*Pb*) or mice infected 5 days earlier with *T*. *brucei* (*Tb*/*Pb*). Cell numbers are presented for the following populations (analyzed within live CD45^+^ cells; n = 4 per group): monocytes/macrophages (CD3^neg^ NK1.1^neg^ CD11b^+^ Ly6G^neg^), neutrophils (CD3^neg^ NK1.1^neg^ CD11b^+^ Ly6G^+^), γδ T cells (CD3^+^ TCRγδ^+^), CD8^+^ or CD4^+^ T cells (CD3^+^ TCRγδ^neg^ NK1.1^neg^ CD8^+^ or CD4^+^, respectively) and NK1.1^+^ cells (CD3^neg/+^ TCRγδ^neg^ NK1.1^+^).
(TIF)

S7 Fig Macrophage and NK/NKT cells are efficiently depleted. **(A-C)** Clec4f, F4/80 and CD68 gene expression quantification by qRT-PCR in the liver 6 h after injection of 3 x 10^4^ *P*. *berghei* sporozoites into naïve mice (*Pb*–blue bars) or mice infected 5 days earlier with *T*. *brucei* (*Tb/Pb*–green bars), non (solid)- or clodronate (patterned)-treated 48 h prior to *P*. *berghei* infection. Bars represent the mean values of four independent experiments and error bars indicate the SEM. Mann-Whitney test was employed to assess the statistical significance of differences between experimental groups. ** *P* < 0.001 and **** *P* < 0.0001. **(D)** Representative plots of flow cytometry gating strategy to analyze NK/NKT cells. **(E)** Assessment of NK/NKT depletion efficiency by flow cytometry in the liver 6 h after injection of 3 x 10^4^ *P*. *berghei* sporozoites into naïve mice (*Pb*–blue) or mice infected 5 days earlier with *T*. *brucei* (*Tb/Pb*–green), injected (open circles) or not (solid circles) with anti-NK1.1 antibody. Results represent the mean values of one representative experiment out of two independent experiments and error bars indicate the SEM. Mann-Whitney test was employed to assess the statistical significance of differences between experimental groups. ns, not significant and ** *P* < 0.01.
(TIF)

S8 Fig The absence of specific subsets of immune cells affects *P. berghei* liver infection. *P*. *berghei* liver infection load quantification by qRT-PCR 6 h after injection of 3 x 10^4^ *P*. *berghei* sporozoites into wild-type, macrophage depleted, *RAG2*^-/-^, *TCRβ*^-/-^, *TCRδ*^-/-^, *JHT*^-/-^, NKT depleted and *IFN-γ*^-/-^ mice, either naïve (*Pb*–blue bars) or infected 5 days earlier with *T*. *brucei* (*Tb*/*Pb*–green bars). Bars represent the mean values of each experimental group normalized to *P*. *berghei*-infected wild-type controls, of two to three independent experiments, with error bars indicating the SEM. The Mann-Whitney test was employed to assess the statistical significance of differences between the experimental groups. ns, not significant, * *P* < 0.05, *** *P* < 0.001 and **** *P* < 0.0001.
(TIF)

S9 Fig *T. brucei*-mediated protection against *P. berghei* liver infection is progressively lost as trypanosomes are eliminated from circulation. **(A)** Left: *P*. *berghei* liver infection load quantification by qRT-PCR 6 h after injection of 3 x 10^4^ *P*. *berghei* sporozoites into untreated (solid bars) or berenil-treated (patterned bars) mice, either naïve (blue bars), or infected 5 or 8 days earlier with *T*. *brucei* (green bars). Right: treatment and infections schedule. Berenil was administered to mice 4 days after *T*. *brucei* inoculation and mice were subsequently infected with *P*. *berghei* sporozoites 1 or 4 days after berenil treatment. Bars represent the mean values of 5 mice from one independent experiment and error bars indicate the SEM. **(B)** Quantification of IFN-γ gene expression by qRT-PCR in the liver 6 h after injection of 3 x 10^4^ *P*. *berghei* sporozoites into untreated (solid bars) or berenil-treated (patterned bars) mice, either naïve (blue bars), or infected 5 or 8 days earlier with *T*. *brucei* (green bars). Bars represent the mean values of 5 mice from one independent experiment and error bars indicate the SEM.
(TIF)

S10 Fig IFN-γ levels positively correlate with the impairment of *P. berghei* liver infection. **(A)** Quantification of IFN-γ gene expression by qRT-PCR in the liver 6 h after injection of 3 x 10^4^ *P*. *berghei* sporozoites into wild-type, *RAG2*^-/-^, *TCRβ*^-/-^, *TCRδ*^-/-^, *JHT*^-/-^ and NKT depleted mice, either naïve (*Pb*–blue bars) or infected 5 days earlier with *T*. *brucei* (*Tb*/*Pb*–green bars). Bars represent the mean values of two to three independent experiments and error bars indicate the SEM. **(B)** qRT-PCR- based quantification of *P*. *berghei* liver infection load 6 h after injection of 3 x 10^4^ *P*. *berghei* sporozoites into naïve or IFN-γ-treated *RAG2*-/- mice. Bars represent the mean values of two independent experiments and error bars indicate the SEM. For **A** and **B**, the Mann-Whitney test was employed to assess the statistical significance of differences between experimental groups. ns, not significant, * *P* < 0.05, ** *P* < 0.01.
(TIF)
