# Supplementary figures and images for: Trypanosoma brucei infection protects mice against malaria
Source: PLoS Pathog. 2019 Nov 8;15(11):e1008145. doi: 10.1371/journal.ppat.1008145 (PMC6867654; doi:10.1371/journal.ppat.1008145)

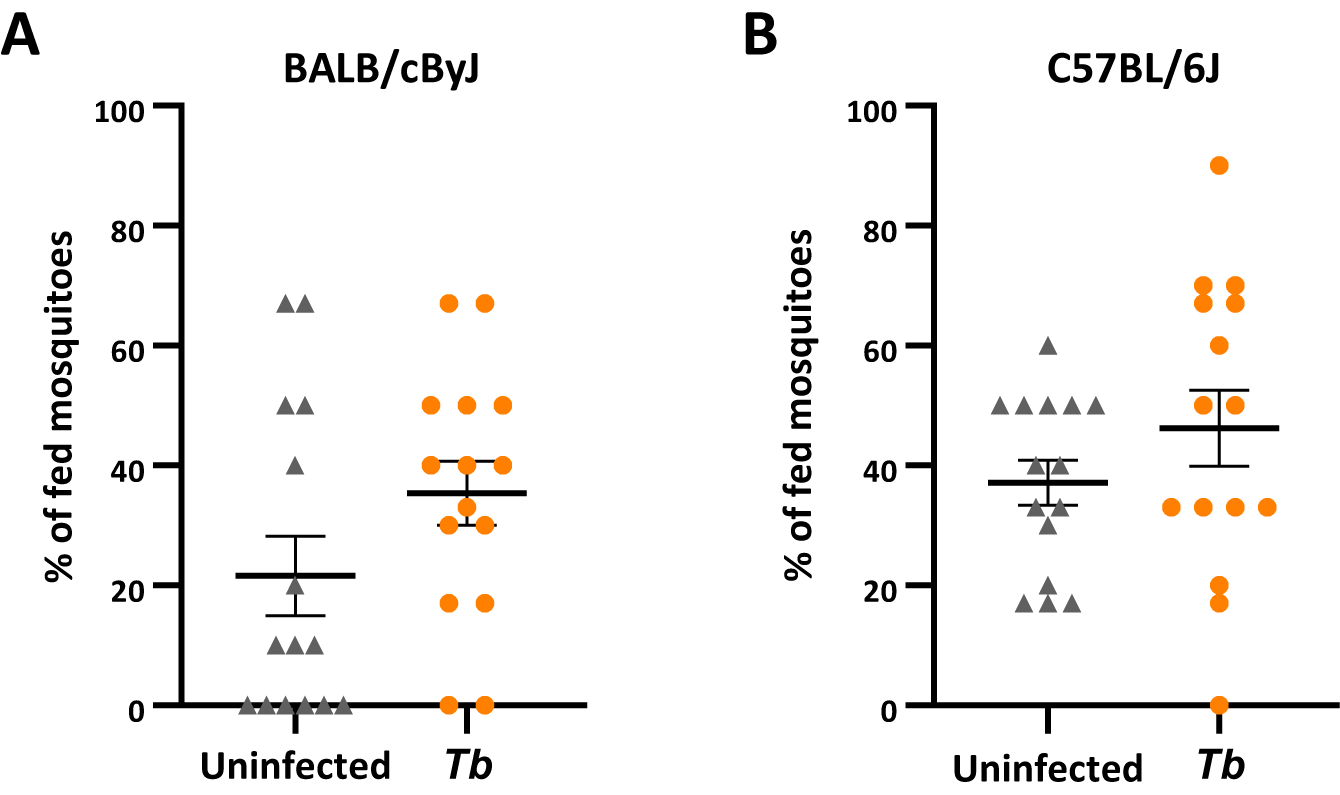

Supplement: S1 Fig — Assessment of mosquito feeding on uninfected or T. brucei-infected BALB/cByJ (A) or C56BL/6J (B) mice. Percentage of mosquitoes that ingested a blood meal and SEM of the pooled data of 15 mice from three independent experiments for each mouse strain are shown. Each dot represents one mouse exposed to the bites of 6 (1 experiment per mouse strain) or 10 (2 experiments per mouse strain) P. berghei-infected mosquitoes. (TIF) [file ppat.1008145.s001.tif]

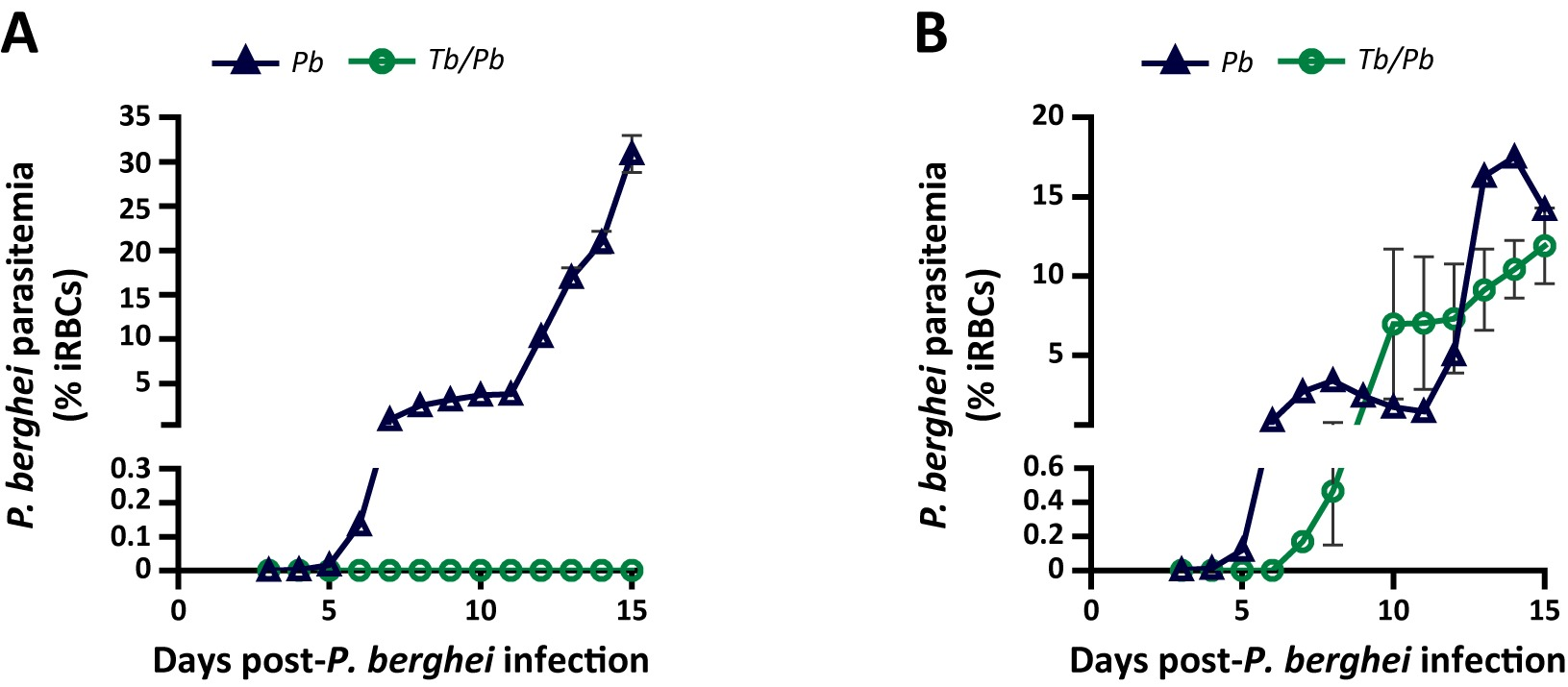

Supplement: S2 Fig — (A) Assessment of P. berghei parasitemia by flow cytometry after inoculation of 500 sporozoites into naïve BALB/cByJ mice (Pb—blue line) or BALB/cByJ mice infected 5 days earlier with T. brucei (Tb/Pb—green line). Percentage of iRBCs and SEM of the pooled data of 10 mice from two independent experiments is shown. (B) Assessment of P. berghei parasitemia by flow cytometry after inoculation of 500 sporozoites into naïve C57BL/6J mice (Pb—blue line) or C57BL/6J mice infected 5 days earlier with T. brucei (Tb/Pb—green line). Percentage of iRBCs and SEM of the pooled data of 10 mice from two independent experiments are shown. (TIF) [file ppat.1008145.s002.tif]

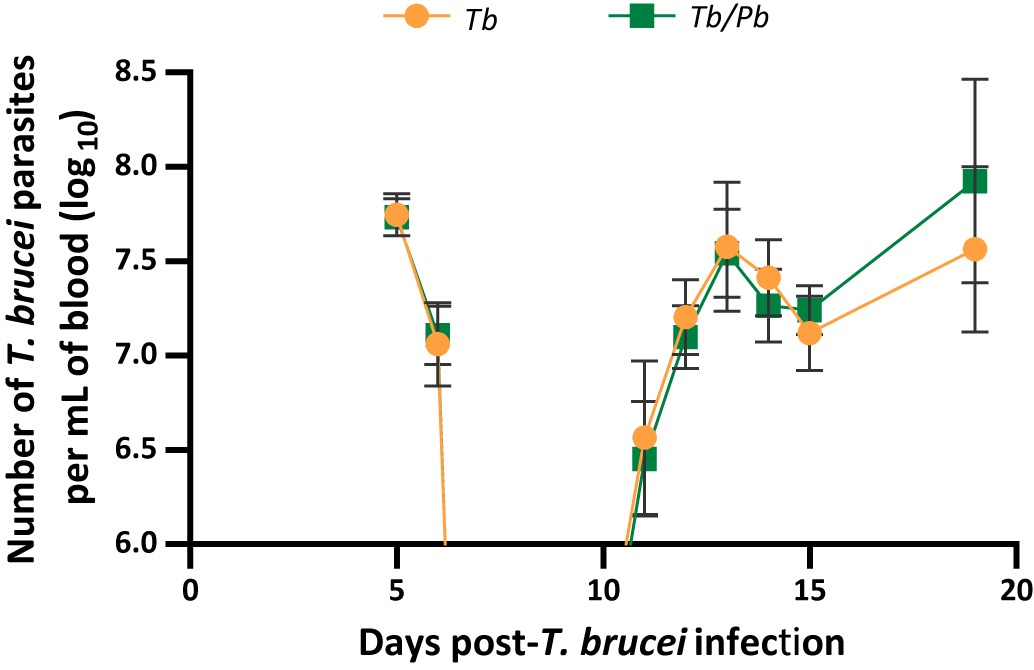

Supplement: S3 Fig — Optical microscopy-based assessment of T. brucei parasitemia in C57BL/6J mice infected only with trypanosomes (Tb–light green line) or inoculated with 1 x 10^6^ P. berghei-iRBCs 5 days after infection with T. brucei (Tb/Pb–dark green line). The geometrical means of the number of trypanosomes per ml of blood and SD of the pooled data of 5 mice from one experiment are shown. (TIF) [file ppat.1008145.s003.tif]

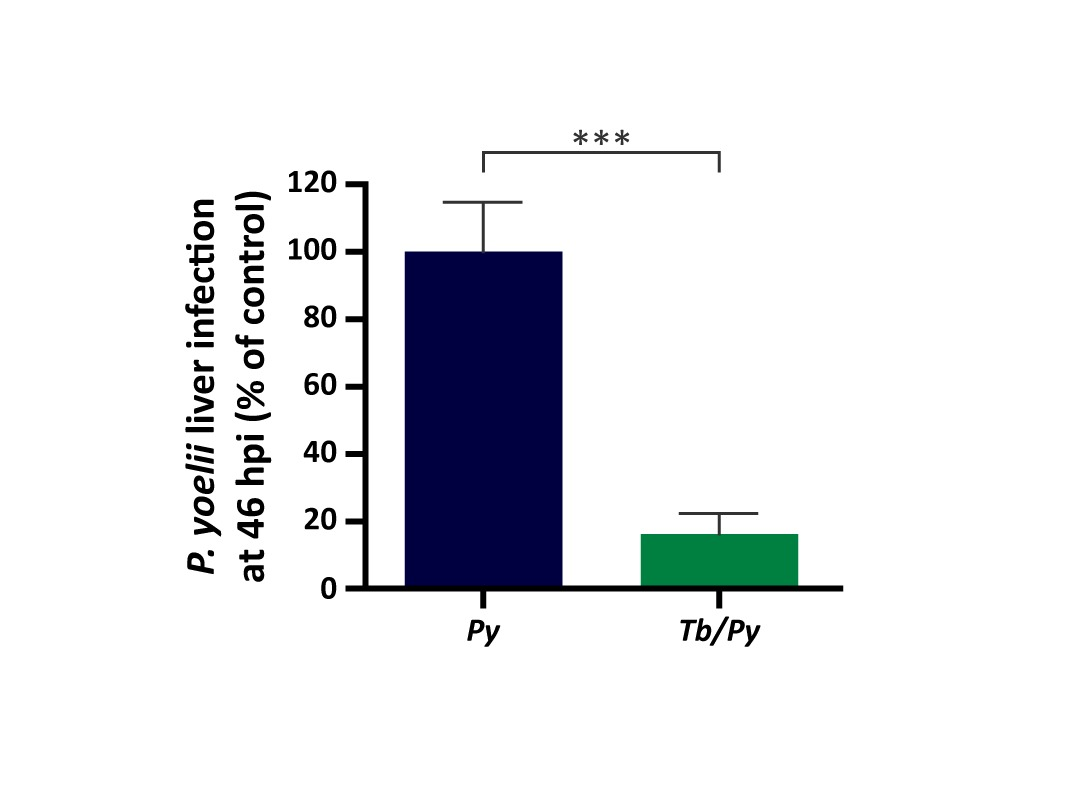

Supplement: S4 Fig — P. yoelii liver infection load determined by qRT-PCR 46 h after sporozoite injection into naïve mice (blue bar) or mice previously infected by T. brucei (green bar). Bars represent the mean values of two independent experiments and error bars indicate the SEM. The Mann-Whitney test was employed to assess the statistical significance of differences between experimental groups (*** P < 0.001). (TIF) [file ppat.1008145.s004.tif]

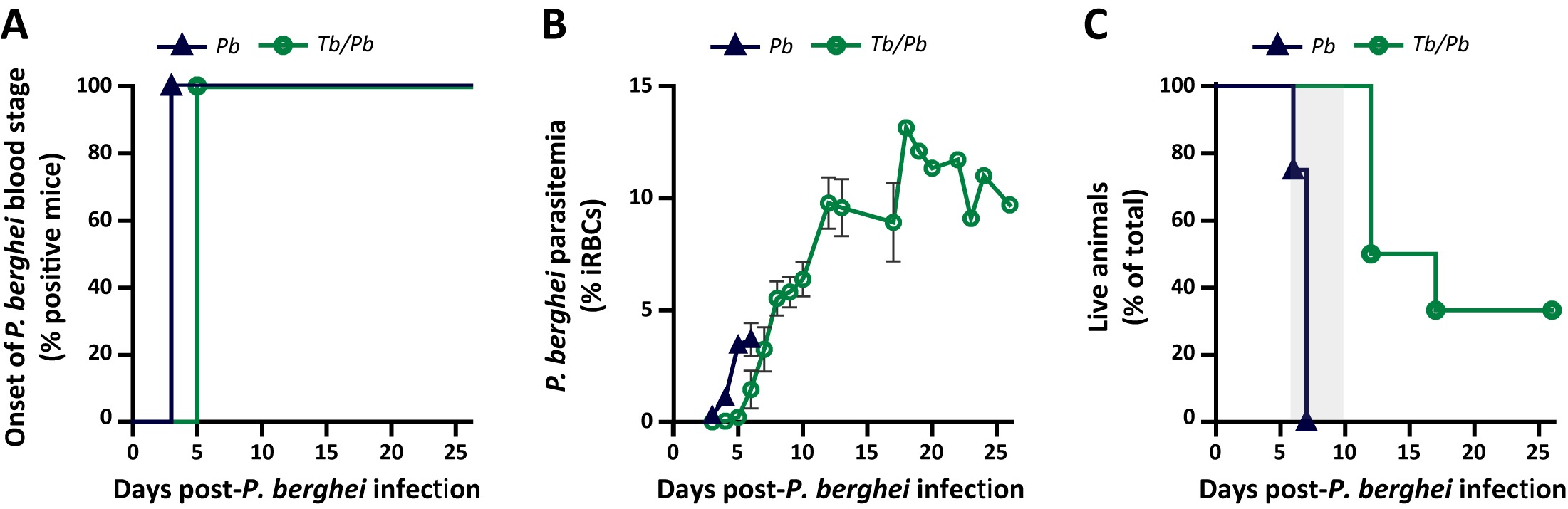

Supplement: S5 Fig — (A) Assessment of P. berghei prepatency period following inoculation of 3 x 10^4^ P. berghei sporozoites into naïve C57BL/6J mice (Pb - blue line) or C57BL/6J mice infected 15 days earlier with T. brucei (Tb/Pb - green line). Percentage of mice displaying P. berghei parasitemia, as measured by flow cytometry. The pooled data of 4–6 mice from one independent experiment is shown. (B) Assessment of P. berghei parasitemia by flow cytometry after inoculation of 3 x 10^4^ GFP-expressing P. berghei sporozoites into naïve C57BL/6J mice (Pb–blue line) or C57BL/6J mice infected 15 days earlier with T. brucei (Tb/Pb–green line). Percentage of iRBCs and SEM of the pooled data of 4–6 mice from one independent experiment are shown. (C) Mouse survival following inoculation of 3 x 10^4^ P. berghei sporozoites into naïve C57BL/6J mice (Pb–blue line) or C57BL/6J mice infected 15 days earlier with T. brucei (Tb/Pb–green line). The pooled data of 4–6 mice from one independent experiment is shown. Time window for ECM development is depicted by the grey-shaded area. (TIF) [file ppat.1008145.s005.tif]

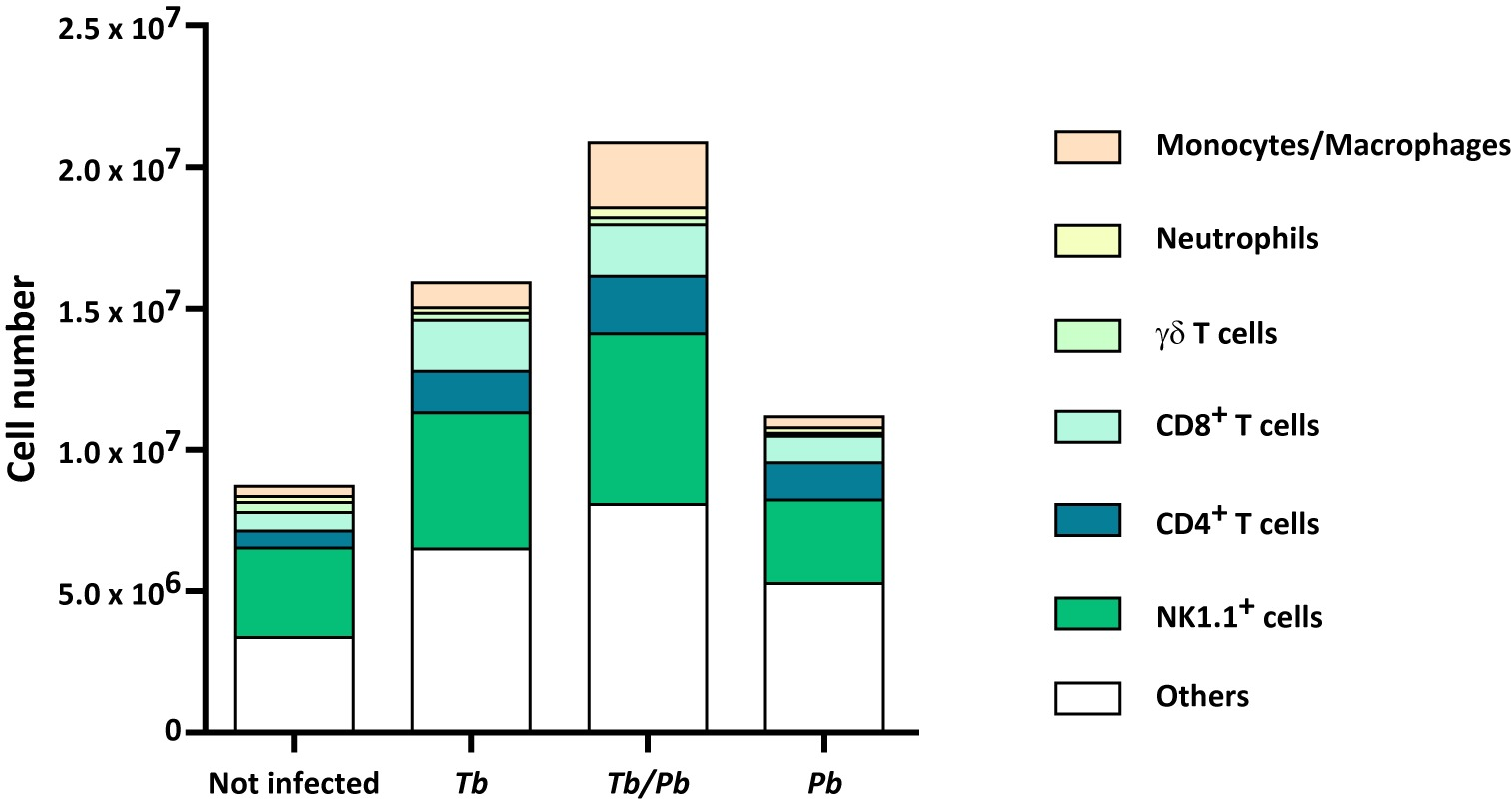

Supplement: S6 Fig — Multi-parameter flow cytometry-based quantification of leukocytes in the liver of mice not infected, infected for 5 days with T. brucei (Tb), co-infected with P. berghei and T. brucei (Tb/Pb), or infected only with P. berghei (Pb). Livers were collected 6 h after injection of 3 x 10^4^ P. berghei sporozoites into naïve mice (Pb) or mice infected 5 days earlier with T. brucei (Tb/Pb). Cell numbers are presented for the following populations (analyzed within live CD45+ cells; n = 4 per group): monocytes/macrophages (CD3neg NK1.1neg CD11b+ Ly6Gneg), neutrophils (CD3neg NK1.1neg CD11b+ Ly6G+), γδ T cells (CD3+ TCRγδ+), CD8+ or CD4+ T cells (CD3+ TCRγδneg NK1.1neg CD8+ or CD4+, respectively) and NK1.1+ cells (CD3neg/+ TCRγδneg NK1.1+). (TIF) [file ppat.1008145.s006.tif]

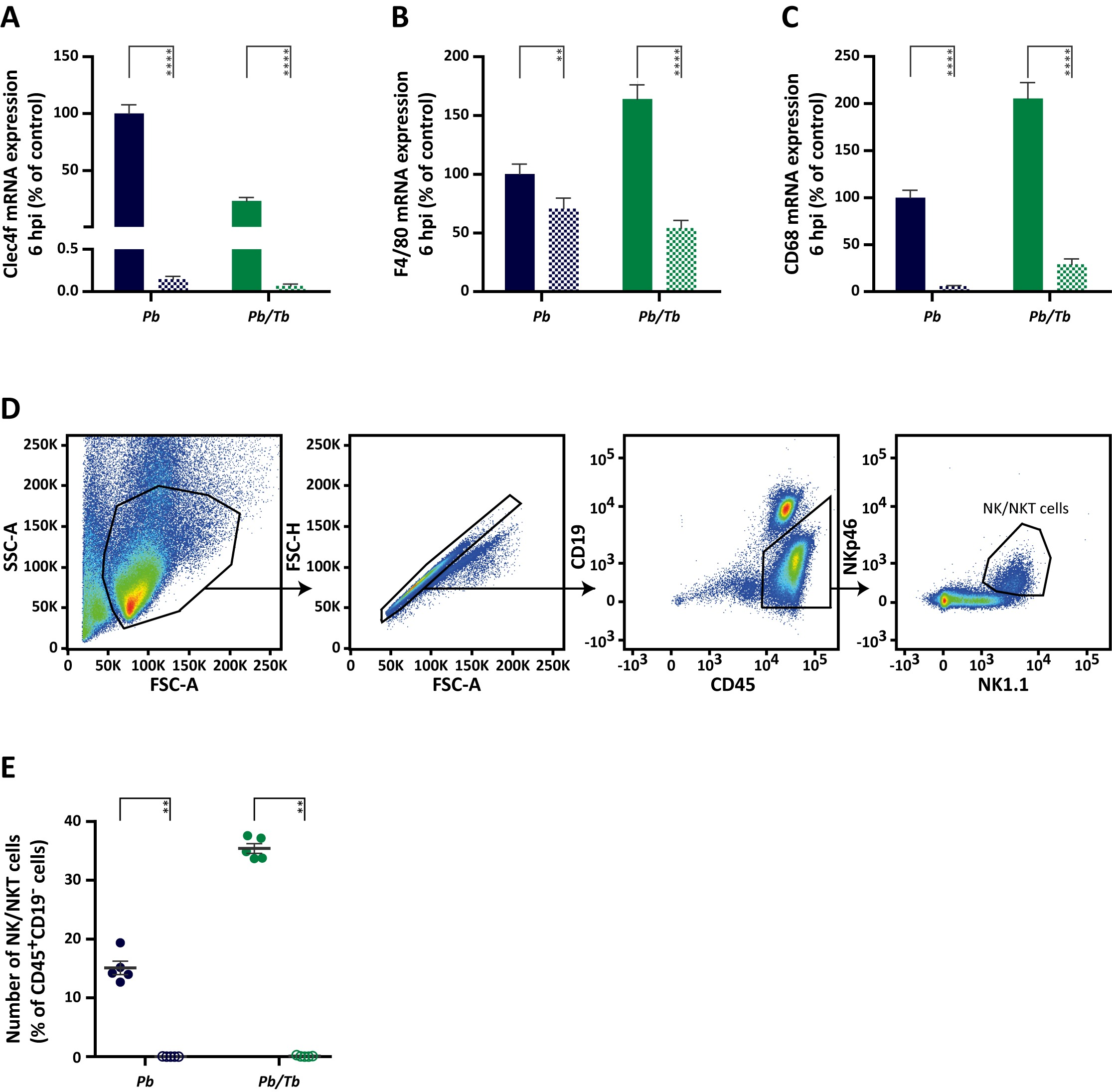

Supplement: S7 Fig — (A-C) Clec4f, F4/80 and CD68 gene expression quantification by qRT-PCR in the liver 6 h after injection of 3 x 10^4^ P. berghei sporozoites into naïve mice (Pb–blue bars) or mice infected 5 days earlier with T. brucei (Tb/Pb–green bars), non (solid)- or clodronate (patterned)-treated 48 h prior to P. berghei infection. Bars represent the mean values of four independent experiments and error bars indicate the SEM. Mann-Whitney test was employed to assess the statistical significance of differences between experimental groups. ** P < 0.001 and **** P < 0.0001. (D) Representative plots of flow cytometry gating strategy to analyze NK/NKT cells. (E) Assessment of NK/NKT depletion efficiency by flow cytometry in the liver 6 h after injection of 3 x 10^4^ P. berghei sporozoites into naïve mice (Pb–blue) or mice infected 5 days earlier with T. brucei (Tb/Pb–green), injected (open circles) or not (solid circles) with anti-NK1.1 antibody. Results represent the mean values of one representative experiment out of two independent experiments and error bars indicate the SEM. Mann-Whitney test was employed to assess the statistical significance of differences between experimental groups. ns, not significant and ** P < 0.01. (TIF) [file ppat.1008145.s007.tif]

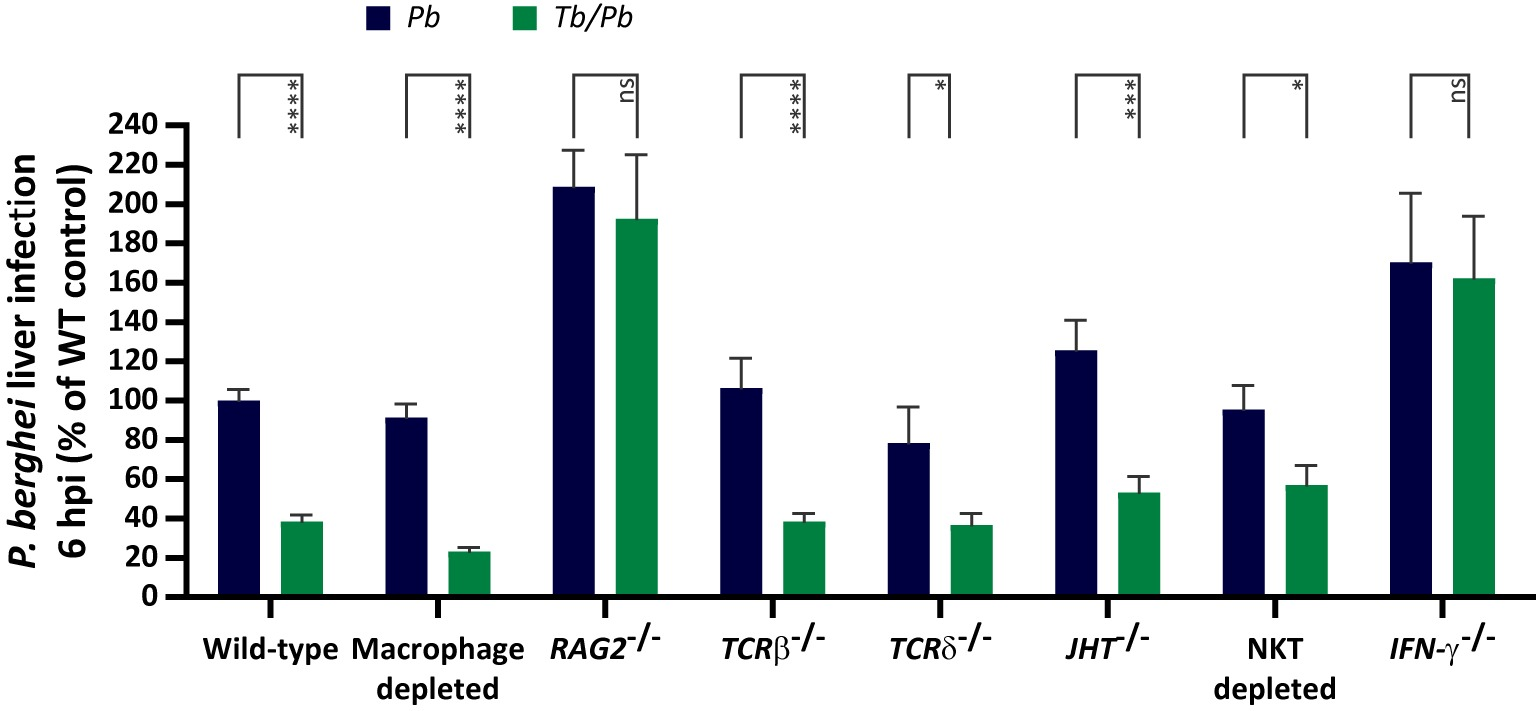

Supplement: S8 Fig — P. berghei liver infection load quantification by qRT-PCR 6 h after injection of 3 x 10^4^ P. berghei sporozoites into wild-type, macrophage depleted, RAG2-/-, TCRβ-/-, TCRδ-/-, JHT-/-, NKT depleted and IFN-γ-/- mice, either naïve (Pb–blue bars) or infected 5 days earlier with T. brucei (Tb/Pb–green bars). Bars represent the mean values of each experimental group normalized to P. berghei-infected wild-type controls, of two to three independent experiments, with error bars indicating the SEM. The Mann-Whitney test was employed to assess the statistical significance of differences between the experimental groups. ns, not significant, * P < 0.05, *** P < 0.001 and **** P < 0.0001. (TIF) [file ppat.1008145.s008.tif]

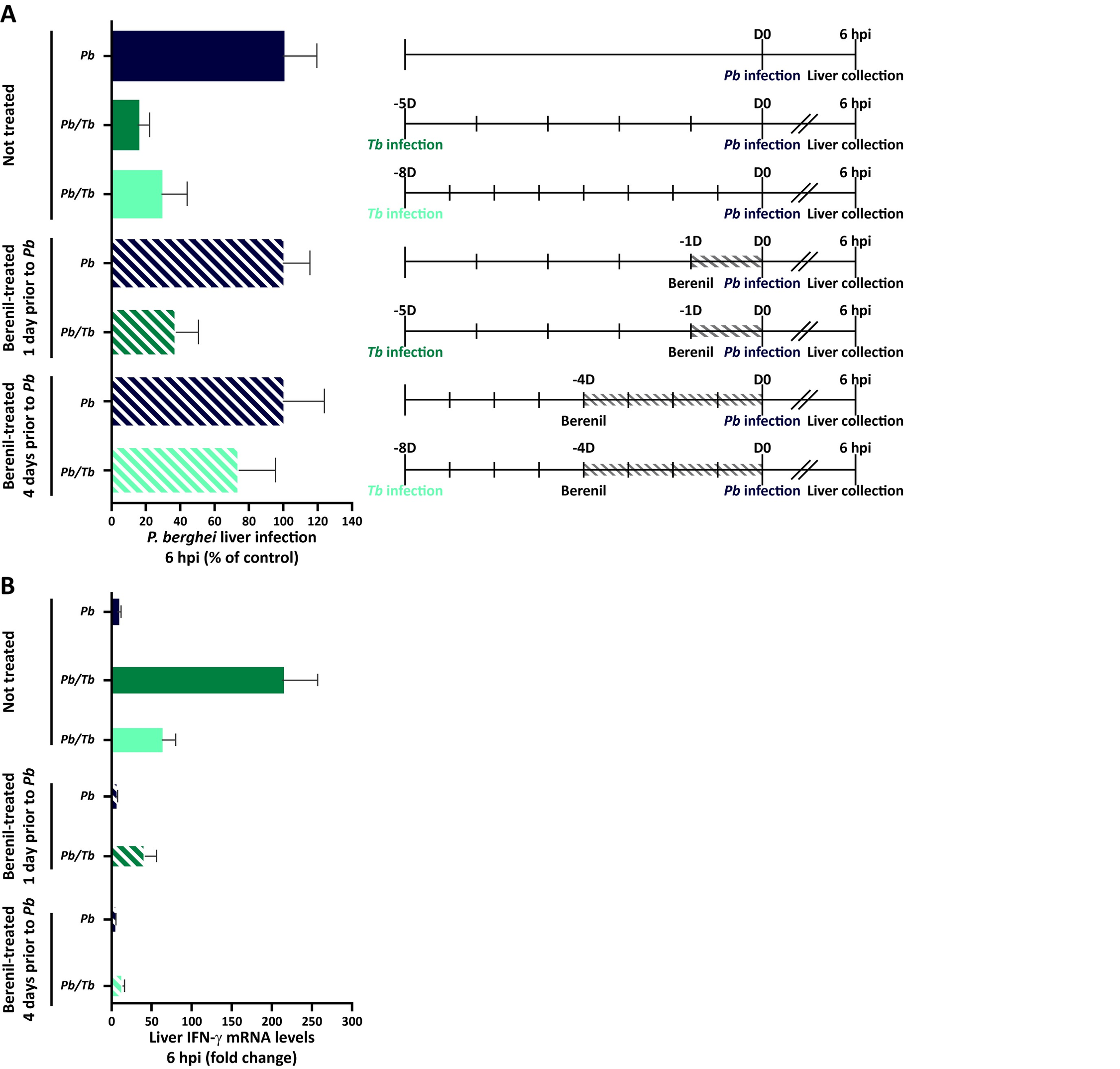

Supplement: S9 Fig — (A) Left: P. berghei liver infection load quantification by qRT-PCR 6 h after injection of 3 x 10^4^ P. berghei sporozoites into untreated (solid bars) or berenil-treated (patterned bars) mice, either naïve (blue bars), or infected 5 or 8 days earlier with T. brucei (green bars). Right: treatment and infections schedule. Berenil was administered to mice 4 days after T. brucei inoculation and mice were subsequently infected with P. berghei sporozoites 1 or 4 days after berenil treatment. Bars represent the mean values of 5 mice from one independent experiment and error bars indicate the SEM. (B) Quantification of IFN-γ gene expression by qRT-PCR in the liver 6 h after injection of 3 x 10^4^ P. berghei sporozoites into untreated (solid bars) or berenil-treated (patterned bars) mice, either naïve (blue bars), or infected 5 or 8 days earlier with T. brucei (green bars). Bars represent the mean values of 5 mice from one independent experiment and error bars indicate the SEM. (TIF) [file ppat.1008145.s009.tif]

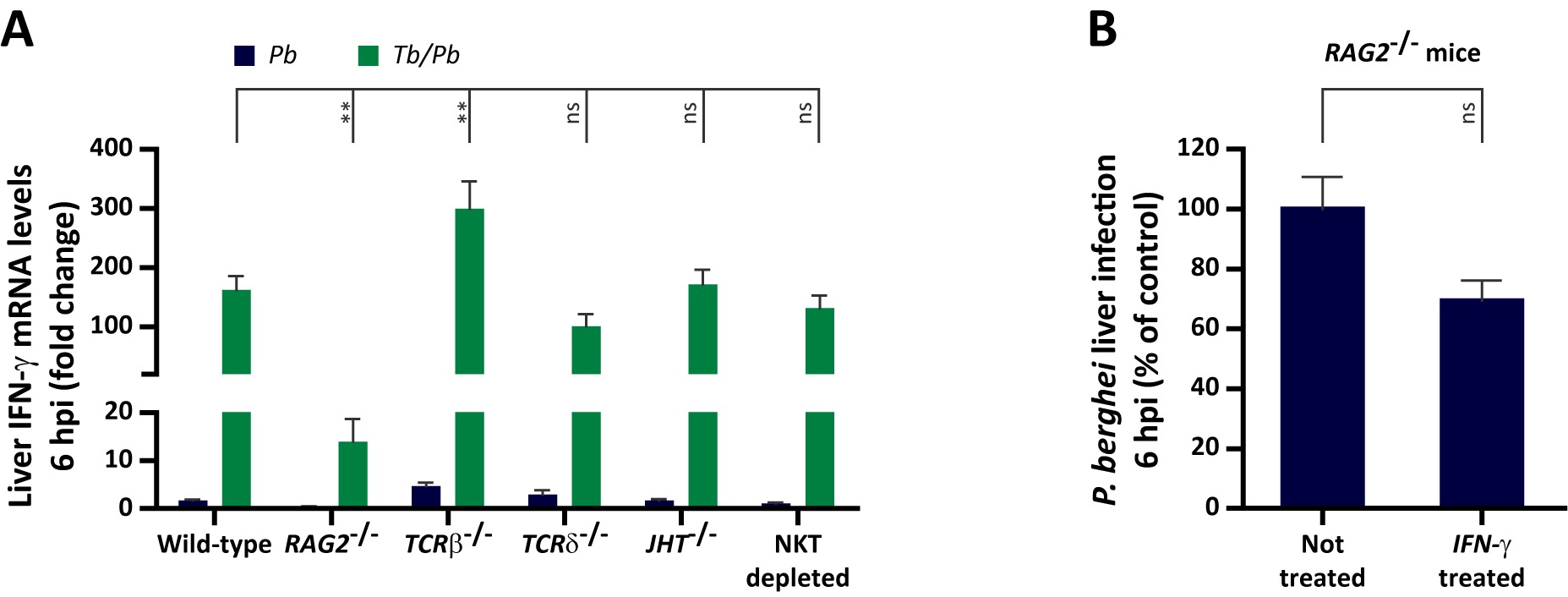

Supplement: S10 Fig — (A) Quantification of IFN-γ gene expression by qRT-PCR in the liver 6 h after injection of 3 x 10^4^ P. berghei sporozoites into wild-type, RAG2-/-, TCRβ-/-, TCRδ-/-, JHT-/- and NKT depleted mice, either naïve (Pb–blue bars) or infected 5 days earlier with T. brucei (Tb/Pb–green bars). Bars represent the mean values of two to three independent experiments and error bars indicate the SEM. (B) qRT-PCR- based quantification of P. berghei liver infection load 6 h after injection of 3 x 10^4^ P. berghei sporozoites into naïve or IFN-γ-treated RAG2-/- mice. Bars represent the mean values of two independent experiments and error bars indicate the SEM. For A and B, the Mann-Whitney test was employed to assess the statistical significance of differences between experimental groups. ns, not significant, * P < 0.05, ** P < 0.01. (TIF) [file ppat.1008145.s010.tif]
